# Transgenerational Propagation and Quantitative Maintenance of Paternal Centromeres Depends on Cid/Cenp-A Presence in *Drosophila* Sperm

**DOI:** 10.1371/journal.pbio.1001434

**Published:** 2012-12-27

**Authors:** Nitika Raychaudhuri, Raphaelle Dubruille, Guillermo A. Orsi, Homayoun C. Bagheri, Benjamin Loppin, Christian F. Lehner

**Affiliations:** 1 Institute of Molecular Life Sciences (IMLS), University of Zurich, Zurich, Switzerland; 2 Centre de Génétique et de Physiologie Moléculaire et Cellulaire, Université Claude Bernard Lyon I, Villeurbanne, France; 3 Institute of Evolutionary Biology and Environmental Studies (IEES), University of Zurich, Zurich, Switzerland; University of Cambridge, United Kingdom

## Abstract

In *Drosophila melanogaster*, as in many animal and plant species, centromere identity is specified epigenetically. In proliferating cells, a centromere-specific histone H3 variant (CenH3), named Cid in Drosophila and Cenp-A in humans, is a crucial component of the epigenetic centromere mark. Hence, maintenance of the amount and chromosomal location of CenH3 during mitotic proliferation is important. Interestingly, CenH3 may have different roles during meiosis and the onset of embryogenesis. In gametes of *Caenorhabditis elegans*, and possibly in plants, centromere marking is independent of CenH3. Moreover, male gamete differentiation in animals often includes global nucleosome for protamine exchange that potentially could remove CenH3 nucleosomes. Here we demonstrate that the control of Cid loading during male meiosis is distinct from the regulation observed during the mitotic cycles of early embryogenesis. But Cid is present in mature sperm. After strong Cid depletion in sperm, paternal centromeres fail to integrate into the gonomeric spindle of the first mitosis, resulting in gynogenetic haploid embryos. Furthermore, after moderate depletion, paternal centromeres are unable to re-acquire normal Cid levels in the next generation. We conclude that Cid in sperm is an essential component of the epigenetic centromere mark on paternal chromosomes and it exerts quantitative control over centromeric Cid levels throughout development. Hence, the amount of Cid that is loaded during each cell cycle appears to be determined primarily by the preexisting centromeric Cid, with little flexibility for compensation of accidental losses.

## Abstract 2

Analysis of centromeres in progeny of *Drosophila* sperm with experimentally altered centromere-specific histone CenH3 levels reveals quantitative inheritance of this epigenetic mark.

## Author Summary

Genetic information in eukaryotic cells is parceled into chromosomes. These information strings are precisely transmitted to daughter cells during mitotic and meiotic cell divisions, but only if the centromere, a specialized chromosomal region, is functional. The centromere region within chromosomes of many species—including humans and the fly *Drosophila melanogaster*—is thought to be specified epigenetically by incorporation of a centromere-specific histone H3 variant (CenH3). After chromosome replication, the centromeres in the resulting two sister chromatids might be expected to be composed of a mixture of pre-existing CenH3 evenly distributed onto the two copies during replication and new CenH3 recruited by the partitioned pool in a stoichiometric manner. Here, we have addressed whether centromeres are indeed replicated in this manner by experimentally altering the levels of centromeric CenH3 in Drosophila sperm. We show that centromeres on paternal chromosomes cannot recruit new CenH3 in embryos fertilized with sperm lacking CenH3. By using sperm with increased or reduced amounts of centromeric CenH3, we demonstrate that altered CenH3 levels are at least partially propagated on paternal centromeres throughout development of the offspring. We conclude that pre-existing CenH3 in Drosophila sperm is therefore not only required for transgenerational centromere maintenance, but that it also exerts quantitative control of this process.

## Introduction

Many eukaryotes, like humans and Drosophila, have chromosomes with a single regional centromere. Faithful propagation of this centromere during chromosome replication and cell proliferation is crucial. Loss of centromere function or extra centromeres cause aneuploidy. Therefore, the molecular mechanisms that control centromere replication have attracted considerable attention recently (for reviews see [1]–[4]). Importantly, these analyses have indicated that centromere identity in regional centromeres is specified epigenetically. Centromere-specific histone H3 variants (CenH3s) are thought to be an essential component of the corresponding epigenetic mark. In humans and Drosophila, the CenH3s have been named CENP-A and Centromere identifier (Cid) (FlyBase accession number FBgn0040477), respectively [5],[6]. Nucleosomes with these CenH3s instead of other histone H3 variants are stably incorporated exclusively within the centromeric region of the chromosome during unperturbed cell cycle progression. The precise structural details of these special centromeric nucleosomes may vary in different cell cycle phases and organisms (reviewed in [1]). Based on the analysis of stretched chromatin fibres, blocks of chromatin containing CenH3 alternate with blocks that lack it [7]. The molecular mechanisms that control the number and size of these blocks and the centromere region overall are not understood. While the gradual depletion of CenH3 does not appear to have immediate effects [8], an enforced acute increase in centromeric Cid has been shown to result in severe chromosome missegregation during mitosis [9].

A conceptually simple mechanism that might maintain the centromere during cell proliferation is “template-governed.” After random distribution of centromeric CenH3 nucleosomes during chromosome replication onto the two sister chromatids, these old nucleosomes may act as a template, allowing the local stoichiometric loading of new CenH3 nucleosomes during each cell cycle. Such a mechanism for maintenance of centromere position and size would lack flexibility for correction of occasional errors. In contrast, “homeostatic” mechanisms controlling the loading of new CenH3s to a target level that is set independently from the actual amount that is already present at the centromere would allow for correction of accidental fluctuations. Elegant experiments in Drosophila have provided clear evidence for template-governed CenH3 loading. Cid-GFP-LacI targeting to lac operator arrays was shown to recruit endogenous Cid that appeared to be maintained independently of Cid-GFP-LacI at least to some extent [10]. On the other hand, recent findings from *C. elegans* and plants have indicated that centromere maintenance during meiosis and onset of embryogenesis can be mechanistically distinct. Cenp-A nucleosomes are transiently eliminated from chromosomes in the *Caenorhabditis elegans* germline and not required for subsequent Cenp-A incorporation in nontranscribed regions throughout the holocentric chromosomes [11],[12]. Although this independence on pre-existing Cenp-A in *C. elegans* might represent a derived state resulting from the evolution of the holocentric chromosomes, a similar transient absence of centromeric CenH3 has also been described in egg cells of *Arabidopsis thaliana* [13], which has regional centromeres. In addition, de novo formation of centromeres can occasionally occur in humans and various experimental systems [14]–[20]. These findings emphasize that in animals, the uncharacterized role of CenH3 in regional centromeres during meiosis and fertilization might not necessarily be the same as during mitotic cell proliferation, where it is both required and sufficient according to the evidence obtained in case of Cid [7],[10],[21]–[23].

**Figure 1:**
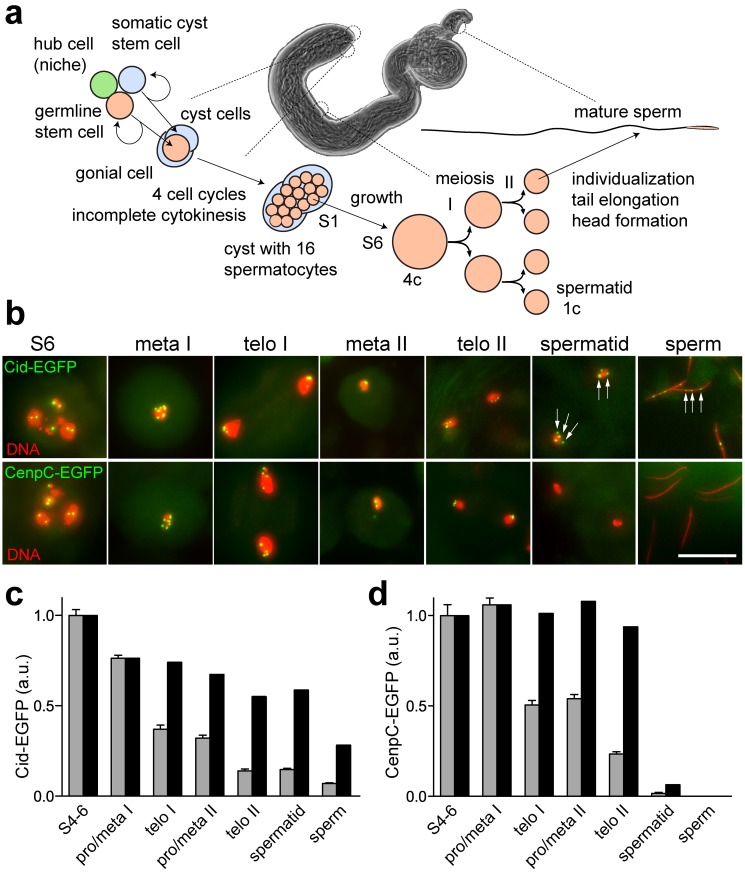
Centromere protein levels during Drosophila spermatogenesis. (a) Schematic overview of spermatogenesis (see also [24]). Spermatocyte stages S1 to S6 as well as the meiotic stages have been described in detail by [25]. (b) Regions from DNA-stained squash preparations of testes expressing either only Cid-EGFP (upper row) or only Cenp-C-EGFP (lower row) instead of endogenous Cid and Cenp-C, respectively, illustrate the stages where EGFP signal intensities were quantified (see panels c and d). White arrows indicate Cid-EGFP signals in postmeiotic stages that lack Cenp-C-EGFP signals. Scale bar, 10 µm. (c and d) Total Cid-EGFP (c) and Cenp-EGFP (d) signal intensity per cell was determined, except for telophase I and II, where each daughter nucleus was analyzed separately. Grey bars represent average intensity in arbitrary units (a.u.), with whiskers indicating s.d. after normalization to the spermatocyte S4–6 value. Black bars indicate centromere protein level per genome equivalent after correction of grey bars according to genome ploidy. Progression through male meiosis is not accompanied by net loading of Cid- and Cenp-C-EGFP onto centromeres, in contrast to mitosis during the syncytial blastoderm [36]. *n*≥20 cells.

To address significance, composition, and transgenerational maintenance of epigenetic centromere marking during sexual reproduction in *Drosophila melanogaster*, we analyzed Cid behavior during spermatogenesis and early embryogenesis. Drosophila spermatogenesis begins at the closed apical end of the testis tube (Figure 1a) [24],[25]. Germline stem cells located there divide asymmetrically. The resulting differentiating daughter cell, the gonioblast, progresses through four mitotic cell cycles with incomplete cytokinesis, and thereby generates a cyst with 16 interconnected spermatocytes. Premeiotic S phase is completed very soon after the last of these four mitotic divisions. Thereafter extensive spermatocyte growth occurs during an extended meiotic G2 phase before progression through the first and second meiotic division. The haploid cell nucleus of postmeiotic spermatids, which remain interconnected within each cyst, is extensively remodeled. Nucleosomes are massively replaced with sperm-specific proteins such as protamines and the genetic material is highly compacted (200-fold) into a needle-shaped sperm head [26]. After complete elongation of the sperm tails, mature sperm is individualized and released in a motile form into the seminal vesicle at the distal end of the testis tube. After fertilization, the sperm nucleus is once more extensively remodeled [27],[28]. Protamines are rapidly replaced with nucleosomes concomitant with transformation into a round male pronucleus. Thereafter progression through the first S phase occurs. In parallel, female meiosis is completed. After S phase and pronuclear migration, the female pronucleus and the closely apposed male pronucleus enter into the first mitosis by forming a gonomeric spindle [29]. The reformation of daughter nuclei in telophase combines the two parental genomes within the first two daughter nuclei. Subsequent progression through the rapid and synchronous cleavage cycles generates a syncytium because cytokinesis is omitted during early Drosophila embryogenesis. After cellularization of the syncytial blastoderm nuclei at the onset of gastrulation, additional cell proliferation involves progression through cell cycles including cytokinesis.

Here we show that Cid survives the radical nucleosome replacement process that accompanies spermatogenesis. Centromeric Cid in sperm also perdures during formation of the male pronucleus after fertilization. Finally, analyses after experimental changes of centromeric Cid levels in sperm demonstrate its crucial role in centromere specification and quantitative maintenance.

## Results

### Paternal Cid But Not Cenp-C Is Inherited with Paternal Centromeres

In case of epigenetic specification of centromere identity, all essential components of the corresponding mark have to be preserved when the bulk of nucleosomes are replaced with protamines during postmeiotic spermatid differentiation. Otherwise paternal chromosomes could not be propagated after fertilization. Cid, the Drosophila CenH3, which is essential for centromere maintenance during mitotic proliferation [21],[22], was therefore expected to be present in mature sperm if Cid is also crucial for transgenerational centromere maintenance. In earlier attempts Cid was not detected in sperm, but technical problems with antigen accessibility during immunolabeling were suspected [30]. To avoid such problems, we analyzed testis from transgenic *cid* mutant males that expressed functional Cid-EGFP under control of the normal *cid* cis-regulatory region instead of endogenous Cid. Specific dot-like EGFP signals were clearly observed in mature *cid; cid-EGFP* sperm (Figure 1b,c), indicating that Cid is indeed present in sperm. While centromeres are strongly clustered close to the chromocenter in most somatic Drosophila interphase cells, Cid-EGFP dots were found to be predominantly unclustered in mature sperm (46%, 42%, and 12% with 4, 3, and 2 signals, respectively; *n* = 24).

In contrast to Cid-EGFP, we were unable to detect Cenp-C-EGFP in mature sperm (Figure 1b,d). During earlier stages, Cenp-C-EGFP was readily detectable (Figure 1b,d). For comparison of Cid and Cenp-C changes during spermatogenesis, centromeric EGFP signal intensities observed in S4–6 spermatocytes were set to 1 arbitrary unit in Figure 1c and d. During the S4–6 stages, however, centromeric Cenp-C-EGFP signals were at least as strong as those observed for Cid-EGFP (unpublished data). Our failure to detect Cenp-C-EGFP in mature sperm is therefore not simply a result of limited detection sensitivity. We conclude that centromeric Cenp-C (FlyBase accession number FBgn0086697) is eliminated during sperm head formation. It is either absent or very low in mature sperm. Another centromere protein described in Drosophila apart from Cid and Cenp-C is Cal1 (FlyBase accession number FBgn0038478) [31]. Cal1-EGFP could also not be detected in sperm (see below). Therefore, Cenp-C and Cal1 do not appear to be essential components of the suspected epigenetic centromere mark.

**Figure 2:**
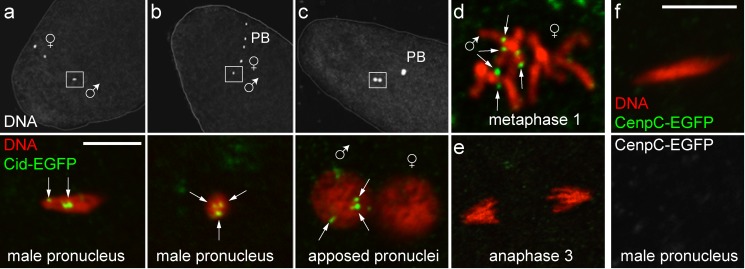
Transmission of paternal Cid to progeny. (a–e) Eggs were collected from females without Cid-EGFP after mating with males with Cid-EGFP. Top panels (a–c) display DNA staining (DNA) at low magnification, and white frames indicate the regions shown at high magnification in the bottom panels. Paternal Cid-EGFP is detected in maximally four spots (white arrows) in the decondensing male pronucleus during (a) and after (b) completion of female meiosis, as well as after pronuclear migration (c). Of 69 male pronuclei analyzed in three independent experiments, 67 were positive for Cid-EGFP. In the gonomeric metaphase plate of the first embryonic mitosis (d), Cid-EGFP is detected on sister centromeres of paternal but not maternal chromosomes. Cid-EGFP is no longer detectable during mitosis 3 (e). (f) In contrast to Cid-EGFP, paternal Cenp-C-EGFP is not transmitted to progeny. It cannot be detected in the decondensing male pronucleus in eggs collected from females without Cenp-C-EGFP after mating with males with Cenp-C-EGFP. None of the analyzed male pronuclei (*n* = 10) and metaphase 1 figures (*n* = 3) displayed detectable GFP dots. PB, polar bodies. Scale bar, 10 µm.

To analyse the fate of paternal Cid protein after fertilization, *cid; cid-EGFP* males were crossed with wild-type females, followed by analyses during the initial cleavage cycles in the resulting embryos. Cid-EGFP signals in up to four discrete spots were readily detected during male pronucleus formation (Figure 2a–c). At metaphase of the first mitosis, Cid-EGFP was present on four pairs of sister centromeres in one of the two chromosome sets within the gonomeric metaphase plate (Figure 2d). Cid-EGFP signals in essentially all of the analyzed paternal pronuclei (11 out of 12) were also observed when males hemizygous for the *cid-EGFP* transgene were crossed to wild-type females. If Cid-EGFP signals in paternal pronuclei, however, were to reflect zygotic expression of the paternally inherited transgene after fertilization, at most 50% of the progeny of hemizygous fathers would be expected to display Cid-EGFP at paternal centromeres. We conclude that the Cid protein of mature sperm remains associated with paternal centromeres during chromatin remodeling and male pronucleus formation, followed by equal distribution onto sister centromeres during the first S phase. During metaphase of mitosis 2, centromeric Cid-EGFP was still detectable but again on only one half of the chromosomes and with reduced intensity (unpublished data). During mitosis 3, paternal Cid-EGFP was no longer detectable (Figure 2e). Progression through the cleavage stages therefore appears to be accompanied by dilution of the inherited paternal Cid-EGFP during each cell cycle by newly recruited unlabeled Cid from maternally provided stores. In contrast to Cid, but as expected from the absence of Cenp-C in mature sperm described above, we did not detect EGFP signals in early embryos after crossing *Cenp-C-EGFP, Cenp-C* males with wild-type females (Figure 2f).

### Sperm Centromere Cid Is Required for Maintenance of Paternal Chromosomes after Fertilization

**Figure 3:**
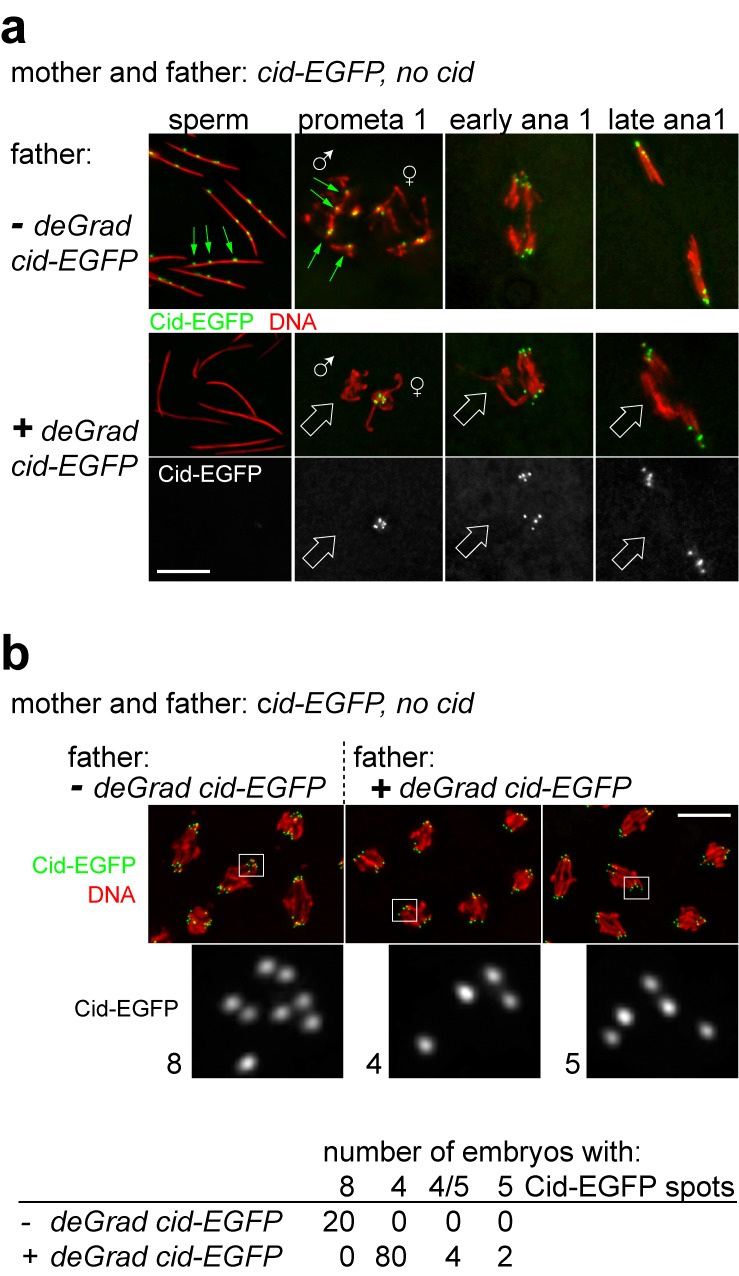
Cid in sperm is required for propagation of paternal chromosomes in progeny. During spermatogenesis, a GFP-specific ubiquitin ligase [32] was either expressed (+ *deGrad cid-EGFP*) or not expressed (− *deGrad cid-EGFP*) in males producing only Cid-EGFP instead of normal Cid. (a) Analysis of their sperm and of early embryos obtained after mating the males with Cid-EGFP females revealed that GFP ubiquitin ligase expression resulted in effective Cid-EGFP depletion in sperm, inhibited maternal Cid-EGFP recruitment onto paternal centromeres, and abolished paternal centromere function during embryonic cycle 1. Centromeric Cid-EGFP signals detectable in − but not + *deGrad cid-EGFP* samples are indicated by green arrows. Chromosomes without Cid-EGFP signals that were not segregated to the poles of mitosis 1 spindles are indicated by white block arrows. (b) Analysis of − and + *deGrad cid-EGFP* progeny during early anaphase of syncytial blastoderm mitoses revealed in each half spindle eight sister centromeres in the former, as expected for diploid embryos, but only four (or rarely five) in the latter. White frames in top panels indicate regions shown at high magnification in bottom panels.

To evaluate the functional significance of paternal Cid inherited with sperm, we applied deGradFP [32] for Cid protein depletion during spermatogenesis. In deGradFP, depletion of GFP fusion proteins is achieved by expression of a GFP-specific recombinant ubiquitin ligase (NSlmb-vhhGFP4) with the UAS/GAL4 system. For expression of this ubiquitin ligase specifically in late spermatocytes, we generated a *topi-GAL4-VP16* driver. Using this driver for deGradFP in *cid; cid-EGFP* males, we were able to obtain sperm in which EGFP signals were no longer above background (Figure 3a). We assume that some centromeric Cid was still present at least during the preceding meiotic divisions, as these were clearly successful. The resulting Cid-depleted sperm allowed successful fertilization, as evidenced by analyses of embryos collected from crosses of deGradFP *cid; cid-EGFP* males with control females. Around 90% of progeny developed to the syncytial blastoderm stage, when thousands of nuclei are regularly arranged just below the egg cell membrane. As fertilization is required for the initiation of embryonic development in *D. melanogaster*, we conclude that fertilization with sperm is still possible after Cid elimination.

However, careful cytological analyses of embryos derived from deGradFP *cid; cid-EGFP* fathers indicated that development after fertilization is not normal. When in control experiments *cid; cid-EGFP* males without deGradFP were crossed to *cid; cid-EGFP* females, we observed normal progeny development with centromeric Cid-EGFP signals in both chromosome sets within all of the analyzed gonomeric metaphase plates of mitosis 1 (Figure 3a; *n* = 10), as expected. However, when deGradFP was active in the *cid; cid-EGFP* males that were crossed to *cid; cid-EGFP* females, one of the two chromosome sets within all of the analyzed gonomeric metaphase plates of mitosis 1 did not display centromeric Cid-EGFP signals (Figure 3a; *n* = 9). This indicates that paternal centromeres cannot acquire maternally derived Cid-EGFP after degradation of Cid-EGFP during spermatogenesis. Mitotic figures in anaphase and telophase of mitosis 1 indicated that Cid-EGFP-free paternal chromosomes did not attach normally to the mitotic spindle. Only the Cid-EGFP containing chromatids were oriented towards the spindle poles in all of the analyzed late mitosis 1 figures (Figure 3a; *n* = 11). We conclude that Cid elimination from sperm results in the loss of paternal chromosomes during the initial syncytial cycles of early embryogenesis.

Gynogenetic haploid embryos obtained from various mutant genotypes (*mh*, *ms(3)K81*, *Hira*) all progress through 14 instead of the normal 13 syncytial blastoderm cycles before cellularization, and they eventually arrest late in embryogenesis [33],[34]. The progeny from *cid; cid-EGFP* fathers with deGradFP expressed these traits as well. First, none of the progeny obtained from these fathers reached the larval stages. We point out that expression of the GFP-specific recombinant ubiquitin ligase (NSlmb-vhhGFP4) with the *topi-GAL4-VP16* driver did not affect male fertility when *cid* function was provided by the endogenous wild-type *cid* gene instead of the *cid-EGFP* transgene. The sterility of *cid; cid-EGFP* fathers with deGradFP therefore does not reflect a Cid-EGFP independent deGradFP effect. Second, compared to progeny derived from wild-type or *cid; cid-EGFP* fathers without deGradFP, the nuclear density during cellularization was 2-fold higher in embryos obtained from *cid; cid-EGFP* fathers with deGradFP (Figure S1).

Counting the number of Cid-EGFP dots during mitosis revealed only four pairs of sister centromeres in the large majority (>90%) of the syncytial blastoderm embryos obtained from a cross of *cid; cid-EGFP* males with deGradFP during spermatogenesis and *cid; cid-EGFP* females (Figure 3b). In contrast, the expected eight pairs of sister centromeres characteristic for the normal diploid karyotype were detected with control fathers lacking deGradFP (Figure 3b).

Centromere counting revealed that a minority (<10%) of progeny from *cid; cid-EGFP* fathers with deGradFP contained nuclei with five pairs of sister centromeres with comparable amounts of Cid-EGFP. Such nuclei were often in patches next to regions with nuclei containing four pairs of sister centromeres. Similarly, a minority of embryos fertilized with Cid-depleted sperm displayed a mosaic of nuclear densities during cellularization with patches of wild-type next to patches with 2-fold higher density (Figure S1), as characteristically observed in near-haploid embryos [35]. While it is not excluded that these near-haploid embryos reflect occasional neocentromere formation or postzygotic centromere restoration by maternal Cid, we favor alternative explanations as discussed below.

### Developmental Regulation of Cid Centromere Loading During Spermatogenesis and Early Embryogenesis

Our analysis of the consequences of Cid-EGFP degradation during spermatogenesis demonstrates that the paternally contributed Cid protein on centromeres of paternal chromosomes is required for normal function of these centromeres. Evidently, the maternally derived Cid supplies present in early embryos cannot be used for restoration of centromere function on paternal chromosomes contributed by Cid-depleted sperm, at least in the great majority of cases. This finding argues against efficient homeostatic compensation of centromeric Cid losses and supports template-governed regulation where Cid recruitment is strictly dependent on already present centromeric Cid. Therefore, the amount of old Cid nucleosomes partitioned onto the two sister chromatids during chromosome replication might determine the loading of a precisely equivalent amount of new Cid into the centromere during cell cycle progression.

Cid recruitment into the centromere occurs during exit from M phase according to our earlier analyses of the syncytial blastoderm cycles [36]. As meiosis includes progression through two consecutive M phases without an intervening S phase, meiotic Cid loading attracted our attention. If new Cid was loaded during both meiotic M phases in amounts precisely equal to the already present centromeric Cid protein, an increase of centromeric Cid levels with each generation had to occur unless compensated by periodic reduction.

**Figure 4:**
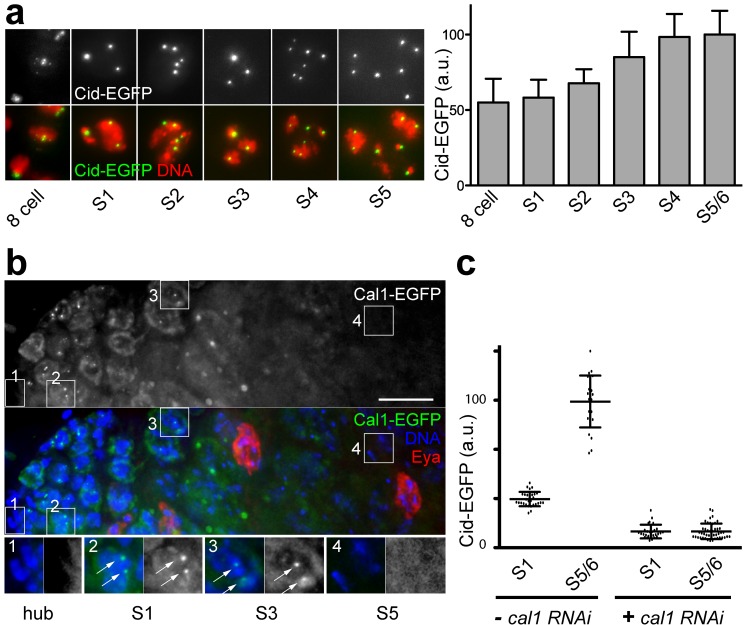
*cal1*-dependent loading of Cid-EGFP during early G2 in spermatocytes. (a) Quantification of EGFP signal intensity per cell revealed an increase in Cid-EGFP levels in spermatocytes between stages S1 and S4. Bars indicate average and whiskers indicate s.d.; *n*>20 cells. (b) Analysis of *cal1-EGFP* expression in testis whole mount preparations indicated that Cal1, which is required for Cid loading during mitotic proliferation [9],[37], is present during the four gonial cycles and during Cid-EGFP loading in early spermatocytes (inset 2, S1; inset 3, S3) but no longer in late spermatocytes (inset 4, S5) and subsequent stages (unpublished data). Cal1-EGFP was also not detectable in postmitotic hub cells (inset 1, hub) and Eya-positive cyst cells. Scale bar, 10 µm. (c) *bamP-GAL4-VP16*-driven expression of a *UAS-cal1RNAi* transgene during late gonial cycles and in early spermatocytes abolished Cid-EGFP loading in early spermatocytes. Dots indicate total Cid-EGFP intensity measured in individual cells. Average intensity (long horizontal line) with s.d. (short horizontal lines) is indicated as well. *n*>24 cells.

To analyse meiotic Cid loading, we quantified centromeric EGFP signals during spermatogenesis in *cid; cid-EGFP* males. Interestingly, this did not reveal any net Cid loading during exit from MI and MII (Figure 1c), suggesting the possibility of compensatory loading during other developmental stages. Indeed, analysis of early spermatocytes revealed net centromeric Cid loading between stage S1 and S4 (Figure 4a)—that is, during G2 well after the premeiotic S phase [25]. The expression pattern of Cal1, a protein required for Cid loading [9],[37], appeared to be entirely consistent with the observed meiotic Cid loading pattern. Cal1-EGFP expressed from a transgene under control of the normal *cal1* cis-regulatory region in a *cal1* null mutant background was detected at centromeres of spermatocytes between S1 and S3 but not during progression through the meiotic divisions (Figures 4b and S2 and unpublished data). Moreover, Cal1 depletion in early spermatocytes by RNAi abolished the increase in Cid-EGFP levels that normally occurred between S1 and S4 (Figure 4c), supporting our conclusion that this Cid-EGFP increase in centromeres of early spermatocytes represents compensatory Cid loading during G2.

**Figure 5:**
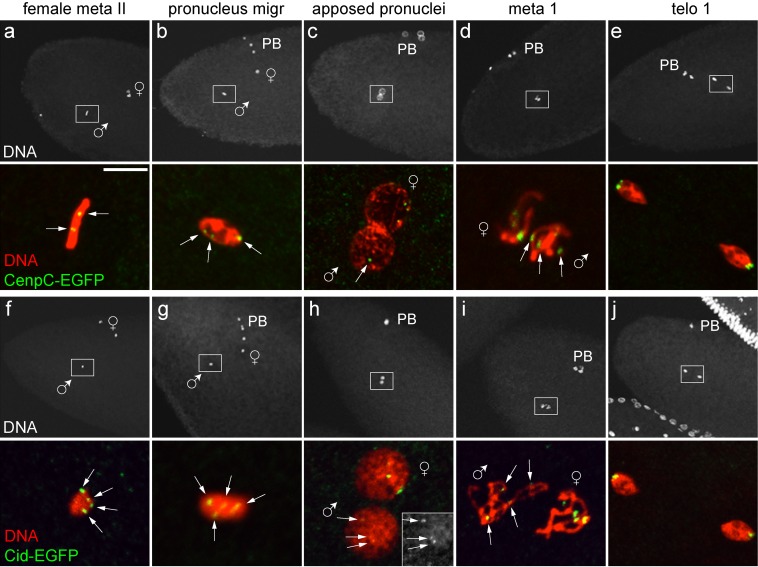
Incorporation of maternal Cid and Cenp-C into paternal centromeres after fertilization. Eggs were collected from transgenic females producing only Cenp-C-EGFP (a–e) or Cid-EGFP (f–j) instead of endogenous Cenp-C and Cid, respectively, after mating with nontransgenic males. The regions indicated by white frames in top panels are shown at high magnification in the bottom panels. (a–e) Maternally derived Cenp-C-EGFP was associated with paternal centromeres (arrows) before full decondensation of the male pronucleus and was present during mitosis 1. (f–j) Maternally derived Cid-EGFP displayed a comparable association dynamic with paternal centromeres (arrows), although signals were generally weaker on paternal centromeres (see h and i). PB, polar bodies. Scale bar, 10 µm.

Apart from Cid loading during spermatogenesis, we also analyzed the initial phase of embryogenesis when sperm nucleus remodeling occurs concomitant with completion of female meiosis. Given that Cenp-C was found to be no longer present on centromeres of mature sperm (see above) and given that this centromere protein provides an essential link between Cid and outer kinetochore components [38],[39], loading of maternally derived Cenp-C onto paternal centromeres during the first cell cycle following fertilization was expected. Therefore, we crossed wild-type males to *Cenp-C-EGFP*; *Cenp-C* females and analyzed progeny during early embryogenesis in order to evaluate whether centromere loading of maternally derived GFP fusion proteins onto paternal centromeres is detectable. Indeed, maternally derived Cenp-C-EGFP was observed to associate very soon after fertilization with the sperm nucleus (Figure 5a,b). Cenp-C-EGFP spots were already observed in sperm nuclei that had not yet attained a regular round shape. Cenp-C-EGFP spots were also present in the paternal pronucleus during S phase and pronuclear apposition (Figure 5c). Moreover, in the first metaphase, Cenp-C-EGFP was present in paternal centromeres just like in the maternal centromeres (Figure 5d,e).

In contrast to Cenp-C, paternal Cid is still present in mature sperm and remains stably associated with paternal centromeres after fertilization, as shown above. Therefore, rapid association of maternally derived Cid before mitosis 1 as in the case of Cenp-C was not necessarily expected. However, in analogous analyses with progeny obtained from *cid; cid-EGFP* mothers and wild-type fathers, such early association of Cid-EGFP was clearly observed (Figure 5f–j). In contrast to the Cenp-C-EGFP experiments, where signal intensities during metaphase 1 were comparable on maternal and paternal centromeres, this was not the case in the Cid-EGFP experiments. Cid-EGFP signal intensities were clearly weaker in paternal compared to maternal centromeres. While both maternal and paternal centromeres contain exclusively the EGFP-tagged version in the Cenp-C experiments, this is only true for the maternal centromeres in case of the Cid experiments, where the paternal centromeres also contain unlabeled wild-type Cid inherited from the father apart from newly loaded maternally derived Cid-EGFP. We conclude that in addition to the net loading of Cid in G2 spermatocytes described above, the rapid association of maternally derived Cid onto paternal centromeres soon after fertilization might provide additional compensation for the absence of Cid loading during the male meiotic divisions. However, we point out that precise quantification of total centromeric Cid-EGFP levels in early embryos is precluded by various factors (like sample thickness, high and variable autofluorescence levels). Thus, we cannot exclude the possibility that the rapid association of maternal Cid-EGFP with paternal centromeres might be balanced by loss of paternal Cid in early embryos. Similarly, we cannot exclude the occurrence of dynamic Cid-EGFP turnover at centromeres during the stages of spermatogenesis where we have not detected any net loading.

### Chromosome-Specific Levels of Centromeric Cid and Kinetochore Proteins

**Figure 6:**
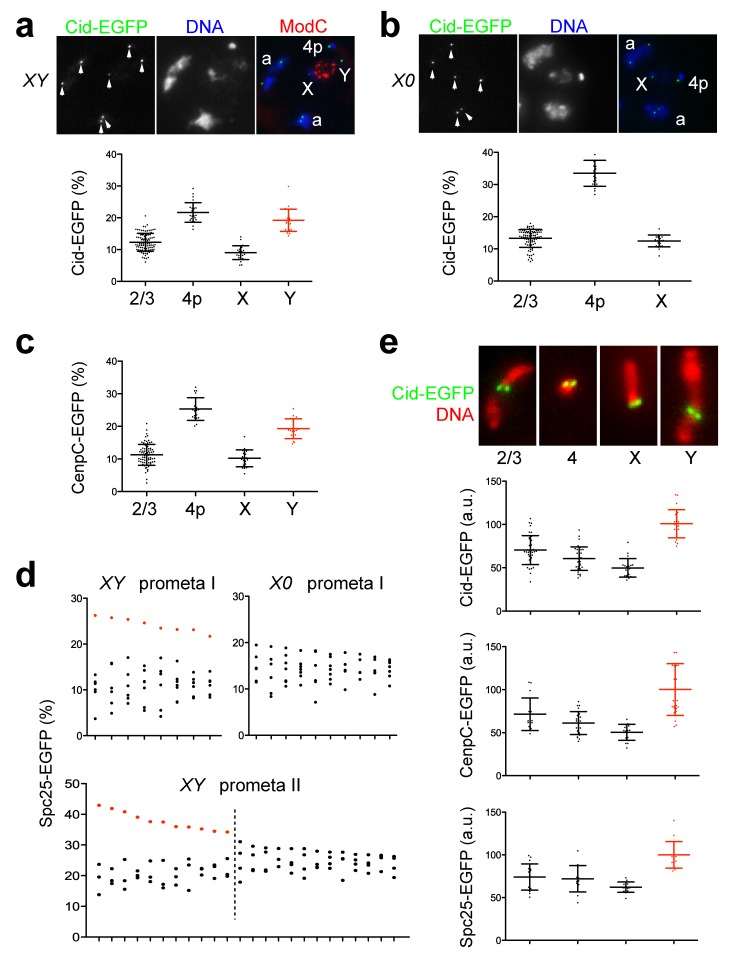
Chromosome-specific differences in centromere and kinetochore protein levels. (a, b) Double labeling of *X/Y*; *cid-EGFP* spermatocytes with anti-ModC (a), which marks the X-Y chromosome territory, and analysis of *X/0*; *cid-EGFP* spermatocytes (b) indicated that the Y centromere contains ∼2-fold higher levels of Cid-EGFP compared to the other centromeres. Dots in the diagrams below the images indicate relative intensity of individual Cid-EGFP dots in S5 stage spermatocytes representing either a chromosome 2 or 3 centromere (2/3), the paired chromosome 4 centromeres (4p), the X centromere (X), or the Y centromere (Y). The sum of all the individually measured centromeric signals within each analyzed spermatocyte was set to 100%. Averages (long horizontal line) are given with s.d. (short horizontal lines). *n*>22. (c) Analogous analysis of *Cenp-C-EGFP* spermatocytes during stage S5 indicated that the Y centromere contains ∼2-fold higher levels of Cenp-C-EGFP compared to the other centromeres. (d) In case of Spc25-EGFP, meiotic cells were analyzed because this kinetochore protein is only present at centromeres during the meiotic M phases. The diagrams display data from cells during prometaphase of meiosis I from either X/Y (XY prometa I) or X/0 (X/0 prometa I) males but only if eight or seven distinct EGFP signals, respectively, could be resolved. In the case of the diagram of prometaphase II in X/Y males (XY prometa II) exclusively, cells with four distinct signals are displayed. Dots in the diagrams below the images indicate relative intensity of individual Spc25-EGFP spots after setting the sum of all the individually measured kinetochore signals within each analyzed cell to 100%. Each column of dots represents one of the analyzed cells. Red dots indicate the values proposed to correspond to the Y centromere. (e) Spreads of mitotic chromosomes were prepared from syncytial blastoderm embryos expressing Cid-EGFP, Cenp-C-EGFP, or Spc25-EGFP and stained for DNA. As illustrated by the image panels, individual chromosomes could be identified based on chromosome size, pattern of intensely staining heterochromatin blocks, and centromere position. Dots in the diagram indicate total centromeric EGFP intensity per chromosome in arbitrary units (a.u.) chosen to result in an average intensity on the Y chromosome of 100 a.u. Averages (long horizontal line) are given with s.d. (short horizontal lines). *n*>15 chromosomes.

By a more detailed quantification of Cid levels during spermatogenesis we addressed yet another aspect of the control of centromeric Cid levels—that is, chromosome-specific variation. Drosophila testis provides a unique advantage for the analysis of chromosome-specific variation of centromeric Cid because of the characteristic segregation of chromosome bivalents into discrete subnuclear territories in late spermatocytes [25]. In principle, an observation of reproducible chromosome-specific differences in centromeric Cid amounts would argue in favor of template-governed control of centromeric Cid levels. Such control would readily propagate distinct chromosome-specific amounts of centromeric Cid. In contrast, homeostatic mechanisms might be expected to equalize occasional fluctuations and keep a uniform level of Cid in all of the centromeres. Therefore, to evaluate whether centromeric Cid amounts vary on different chromosomes, we quantified EGFP signals in individual centromeres of S5/6 spermatocytes in *cid; cid-EGFP* testis preparations. At the S5/6 stage, DNA staining revealed the three characteristic chromosome territories within the large spermatocyte nucleus. Two of these territories represent the bivalents of chromosome 2 and 3, respectively. Their DNA labeling is more homogenous than that of the third territory, which is formed by an association of the bivalent of chromosome 4 with the X chromosome and those parts of the Y that are not involved in Y loop formation [25]. The territories with the bivalents of chromosome 2 and 3 both contained two Cid-EGFP spots (Figure 6a). Each spot is known to represent the tightly associated sister centromeres of one homolog [40]. Double labeling with anti-ModC [41],[42] allowed the identification of the X-Y bivalent (Figure 6a). The X-Y region was observed to be associated with two spots of obviously unequal Cid-EGFP intensity. An additional bright spot was usually observed in close association with a dot of very bright DNA staining near the X-Y region (Figure 6a). This bright Cid-EGFP spot represents the paired centromeres of the small dot-like chromosome 4 bivalent.

The characteristic unequal intensity of the two Cid-EGFP spots within the X-Y chromosome territory suggested that either the X or the Y centromere is associated with higher levels of centromeric Cid. To clarify this issue we crossed *cid-EGFP* into X/0 males. Apart from the paired centromeres of chromosome 4, the X/0 spermatocytes no longer contained a second bright Cid-EGFP spot (Figure 6b), as characteristically present in normal X/Y spermatocytes (Figure 6a). Therefore, we conclude that the Y centromere contains more Cid than all the other centromeres. A quantification of the Cid-EGFP signals on the different chromosomes revealed that the Y centromere contains ∼2-fold more Cid than the other centromeres. Analyses with Y chromosomes introgressed from different Drosophila strains into the *cid; cid-EGFP* background indicated that the increased Cid levels on the Y centromere are not strain-specific (Figure S3).

Analogous quantification of Cenp-C revealed that the level of this centromere protein was also ∼2-fold higher on the Y centromere (Figure 6c). To evaluate whether the ∼2-fold higher levels of the centromere proteins Cid and Cenp-C on the Y centromere were accompanied by a corresponding increase in kinetochore components, we analyzed Spc25-EGFP signals. Spc25 is a component of the Ndc80 complex, which represents the major microtubule binding site of the kinetochore. Before the onset of the meiotic divisions, we did not detect dot-like Spc25-EGFP signals. However, during prometaphase of meiosis I, spermatocytes often displayed eight distinct Spc25-EGFP signals, as expected. In such prometaphase I figures, one of the eight signals was always considerably stronger than all the others (Figure 6d). In contrast, in X/0 testis, prometaphase I figures with seven distinct Spc25-EGFP signals did not include such a conspicuously stronger signal (Figure 6d), suggesting that the especially strong Spc25-EGFP signals in X/Y testis represent the Y kinetochore. As predicted by this interpretation, prometaphase II figures in X/Y testis with 4 Spc25-EGFP signals could readily be grouped into two classes: a first class with a conspicuously strong signal, and a second class without such an intensity outlier. In all likelihood, these two classes represent early spermatids that had inherited the Y and the X chromosome, respectively, in the preceding meiosis I. Finally, a quantification of kinetochore signal intensities in mitotic chromosomes released from early syncytial embryos provided a further confirmation that the Y centromere has higher levels of Cid, Cenp-C, and Spc25 (Figure 6e). Thus, the increased levels of centromere and kinetochore proteins on the Y centromere are not a peculiarity of the spermatocyte stages. Moreover, these observations argue against the existence of efficient homeostatic mechanisms that enforce identical Cid amounts on all the different centromeres.

### Transgenerational Propagation of Altered Centromeric Cid Levels in Sperm

For a direct evaluation of the role of centromeric Cid for quantitative maintenance, we generated sperm with either moderately increased or decreased levels of Cid on centromeres and analyzed whether the altered centromeric levels were maintained during development of the next generation.

**Figure 7:**
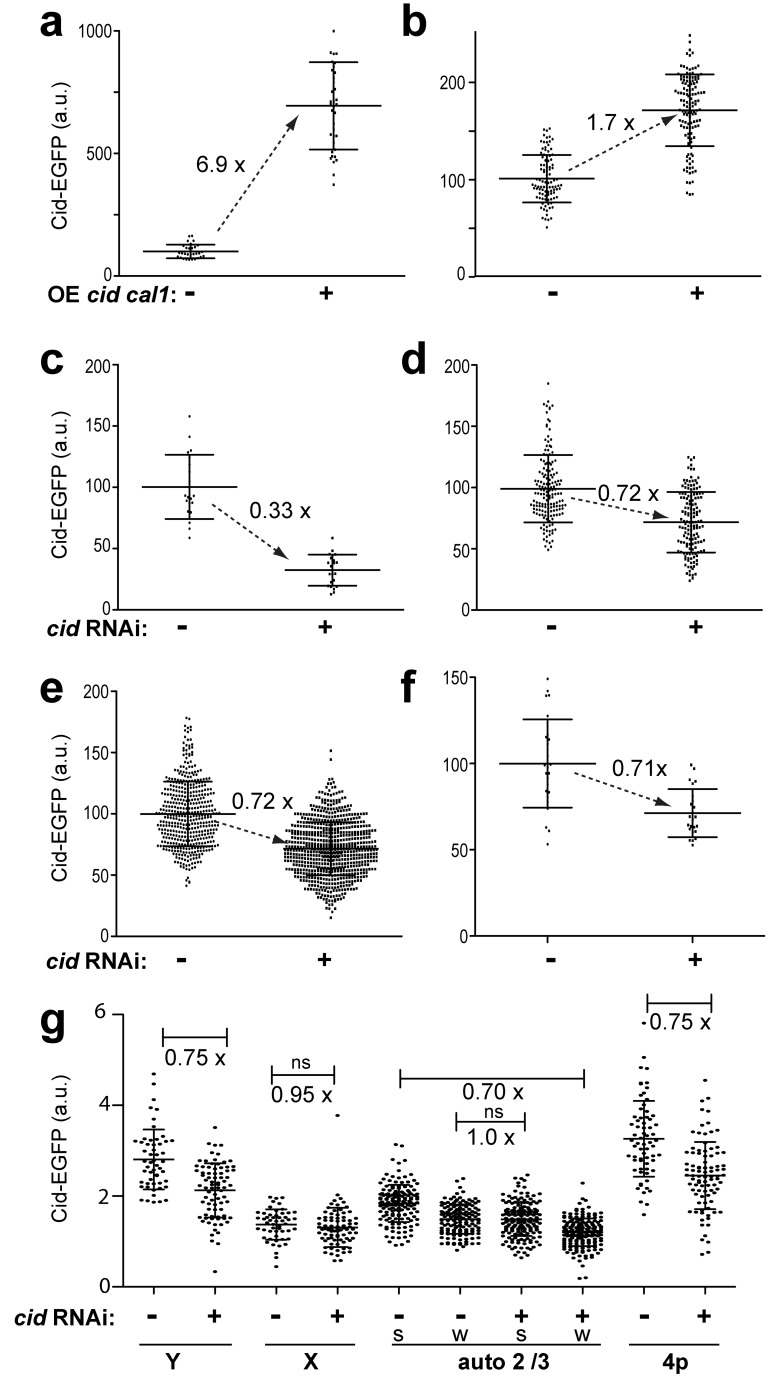
Transgenerational maintenance of Cid levels. Experimentally, centromeric Cid-EGFP levels were either increased (a, b) or decreased (c–g) in sperm in a background producing only Cid-EGFP instead of endogenous Cid. Sperm with altered centromeric Cid-EGFP levels was used for progeny generation. Propagation of altered Cid-EGFP levels during progeny development was analyzed. (a) Comparison of the total amount of Cid-EGFP per sperm in males without (−) or with (+) *bamP-GAL4-VP16*-driven expression of *UAS-cid-EGFP* and *UAS-cal1*. (b) Comparison of the total amount of Cid-EGFP per nucleus in syncytial blastoderm embryos derived from males without (−) or with (+) increased Cid-EGFP in sperm as determined in (a). (c) Comparison of the total amount of Cid-EGFP per sperm in males without (−) or with (+) *bamP-GAL4-VP16*-driven expression of *UAS-Cid^RNAi^*. (d–f) Comparison of the total amount of Cid-EGFP per nucleus in progeny derived from males without (−) or with (+) decreased Cid-EGFP in sperm as determined in (c) at different developmental stages: syncytial blastoderm (d), wing imaginal discs of third instar larvae (e), and sperm of adult males (f). Dots in (a–f) indicate total centromeric EGFP intensity per nucleus in arbitrary units (a.u.) chosen to result in an average intensity of 100 a.u. in the controls where Cid-EGFP was neither increased nor decreased in sperm. Averages (long horizontal line) are given with s.d. (short horizontal lines). *n*>22. The fold change of average Cid-EGFP levels between controls and experimental samples is indicated next to the dashed arrows. All the indicated differences were found to be highly significant (*p*<0.001, *t* test). (g) Comparison of Cid-EGFP levels in individual centromeres of Y (Y), X (X), major autosomes (2/3), and the paired chromosome 4 centromeres (4p) in S5 spermatocytes of adult progeny derived from *P{w^+^, pUASt-mCherry-nls}III* females mated to males without (−) or with (+) decreased Cid-EGFP in sperm as determined in (c). Each major autosome territory contains two Cid-EGFP spots. The stronger (s) and weaker (w) spots, respectively, were grouped and analyzed separately. Dots indicate centromeric EGFP intensity in arbitrary units (a.u.). Averages (long horizontal line) are given with s.d. (short horizontal lines). *n*>50. The fold change of average Cid-EGFP levels between controls and experimental samples is indicated below brackets. The corresponding differences of the averages are highly significant (*p*<0.001, *t* test) except for two nonsignificant cases (*ns*).

To raise centromeric Cid levels in sperm, we used the UAS/GAL4 system for targeted *cid-EGFP* overexpression during spermatogenesis. Overexpression was driven in a *cid; cid-EGFP* background that did not produce any untagged wild-type Cid. Therefore, the accurately quantifiable Cid-EGFP was the only Cid species produced. Concomitantly with *UAS-cid-EGFP*, we also expressed *UAS-cal1* because increased Cid deposition in centromeres was previously found to depend on simultaneous overexpression of *cid* and *cal1* [9]. *bam-GAL4-VP16*-driven co-expression of *UAS-cid-EGFP* and *UAS-cal1* in *cid; cid-EGFP* testis resulted in a strong increase in centromeric Cid-EGFP signals in sperm compared to controls lacking the UAS transgenes (Figure 7a). Quantification revealed almost 7-fold higher Cid-EGFP levels after overexpression. Judging from the number and size of the observed Cid-EGFP spots, Cid-EGFP was still primarily confined to the centromeric region.

Males with “high Cid-EGFP” sperm as well as control males lacking the UAS transgenes were crossed with *cid; cid-EGFP*; *Cenp-C-Tomato* females and progeny was aged to the syncytial blastoderm stage before fixation and quantification of centromeric Cid-EGFP signals in prometa- and metaphase embryos. The total centromeric Cid-EGFP intensity per nucleus was found to be ∼1.7-fold higher in embryos generated with high Cid-EGFP sperm compared to embryos generated with control sperm (Figure 7b). Centromeric Cenp-C-Tomato was increased to a comparable extent (unpublished data). Considering that only one half of the centromeres in the embryo are of paternal origin, we conclude that the increased Cid-EGFP levels on paternal centromeres appear to be maintained during progression through the early embryonic cell cycles, although not quantitatively. The level of Cid-EGFP during embryogenesis might not be sufficiently high to support a complete maintenance of the paternally increased centromeric Cid-EGFP levels during postzygotic development. Results from an analysis of the effects of the zygotic *cid-EGFP* gene dose on centromeric Cid-EGFP levels in wing imaginal disc cells of third instar wandering stage larvae and in spermatocytes of adult males supported the notion that the expression level governed by the normal *cid* regulatory region is not much higher than what is required for maintenance of physiological centromeric Cid levels. In the absence of endogenous Cid, cells with only one *cid-EGFP* copy were observed to display centromeric signals that were 40% weaker than those in cells with two *cid-EGFP* copies (Figure S4).

As limiting *cid* expression might have prevented complete maintenance of increased Cid levels on paternal centromeres, we also analyzed whether decreased Cid levels on paternal centromeres in sperm are maintained during development of the next generation. Transgenic RNAi allowed a partial Cid depletion during spermatogenesis. Targeted depletion using *bam-GAL4-VP16* in combination with a *UAS-cid^RNAi^* transgene was achieved in a *cid; cid-EGFP* background lacking untagged wild-type Cid. Quantification of centromeric signals in sperm indicated that RNAi resulted in a reduction of Cid-EGFP to about 33% of its level in controls lacking the *UAS-cid^RNAi^* transgene (Figure 7c). In a second independent experiment, a somewhat lower reduction to about 50% was obtained, and the centromeres of X, Y, and autosomes were found to be affected to a comparable degree (Figure S5a,b). Males producing low Cid-EGFP sperm and control males were crossed with *cid; cid-EGFP*; *Cenp-C-Tomato* females, and centromeric Cid-EGFP levels in progeny were determined at the syncytial blastoderm stage. The total centromeric Cid-EGFP intensity per nucleus in the embryos derived from low Cid-EGFP sperm was found to be ∼72% of the intensity observed in the controls (Figure 7d). Considering that only one half of the centromeres are of paternal origin, the reduced Cid-EGFP levels on paternal centromeres appeared to have been quantitatively maintained during progression through the early embryonic cell cycles.

To evaluate whether the reduced Cid-EGFP levels were also maintained during subsequent development, we analyzed wing imaginal discs from third instar wandering stage larvae. These measurements revealed that the reduced Cid-EGFP levels were indeed maintained beyond embryogenesis (Figures 7e and S5c,d). Finally, we measured centromeric Cid-EGFP levels in sperm of adult male progeny. As in embryos and imaginal discs, only ∼71% of the control levels were observed in sperm of males fathered by Cid-depleted sperm (Figure 7f).

Since chromosome territory formation in spermatocytes is accompanied by conversion of chromocenter-associated centromere clusters into well-separated centromeres, we were able to quantify Cid-EGFP levels in individual centromeres in this special cell type. Because the X and Y chromosomes are of maternal and paternal origin, respectively, only the Y but not the X centromere is expected to have reduced centromeric Cid-EGFP, if the reduction reflects propagation on paternal centromeres after Cid depletion during spermatogenesis in the father. Indeed reduction of Cid-EGFP in paternal sperm was found to result in a significant decrease of Cid-EGFP in the Y but not in the X centromere in two independent experiments (Figure 7g, and unpublished data).

In case of chromosome 2 and 3 territories, parental origin could not be assigned to the two signals within a territory. Under the assumption that in control spermatocytes Cid amounts in maternally and paternally derived centromeres of chromosome 2 and 3 are usually equal on average, the results obtained after quantification of centromeric Cid-EGFP signal intensities in major autosome territories were not in accord with the findings concerning the X and Y centromeres. Under this assumption it is expected that the intensity difference between the stronger and weaker centromere signal within a major autosome territory should be greater after reduction in sperm and subsequent propagation of reduced Cid on paternal centromeres in comparison to control spermatocytes. However, the average intensity difference between the two signals of a major autosome territory was not increased after reduction of Cid-EGFP in paternal sperm (Figure 7g). In principle, this result might argue for chromosome-specific differences in the control of centromeric Cid levels on sex chromosomes and autosomes. However, this apparent support for chromosome-specific discrepancies is completely abolished under the following alternative assumption. If centromeric Cid levels in control spermatocytes on average are usually higher on paternal compared to maternal centromeres, our results are clearly consistent with quantitative propagation of centromeric Cid not only on the Y but on all paternal centromeres. According to this alternative assumption, the stronger of the two signals in each major autosome territory within control spermatocytes in general corresponds to the paternal and the weaker to the maternal centromere. After reduction in sperm and subsequent propagation of reduced centromeric Cid, only the paternal (i.e., the stronger) but not the maternal (i.e., the weaker) centromere signals should be decreased. This expectation is borne out by our data (Figure 7g). While statistical analyses did not favor one over the other assumption, we propose that our other findings (Figures 5 and 6) provide support for the second assumption, as discussed below.

Our comparison of spermatocytes in males with either two or only one Cid-EGFP gene copies also corroborated the second interpretation. After reduction of the zygotic Cid-EGFP gene dose, centromeric Cid-EGFP was no longer decreased exclusively on the Y centromere (as after centromeric Cid-EGFP reduction in paternal sperm), but equally on both sex chromosomes, and also on all autosomal centromeres (Figure S4C).

Based on our analysis of the consequences after reduction of centromeric Cid in sperm, we conclude that centromeric Cid-EGFP is not replenished to normal levels during development of progeny, at least in case of the Y centromere and presumably also on all other centromeres.

## Discussion

Among the known Drosophila centromere proteins (Cid, Cenp-C, Cal1), only Cid survives the excessive chromatin remodeling that accompanies the compaction of the haploid genome into sperm heads. We demonstrate that this centromeric Cid in sperm is essential for the propagation of the paternal genome in the next generation. When normal oocytes are fertilized with sperm lacking centromeric Cid, paternal chromosomes fail to recruit the maternally provided Cid and cannot generate functional kinetochores during mitosis 1. As a result, gynogenetic haploid embryos develop. These findings demonstrate that a minimal amount of pre-existing centromeric Cid is required for centromere propagation during cell cycle progression. Moreover, by partial depletion of centromeric Cid in sperm, in combination with precise quantification, we establish that pre-existing centromeric Cid not only functions as a permissive factor but actually exerts quantitative control over centromeric Cid maintenance during cell proliferation. Reduced centromeric Cid levels in sperm are maintained throughout development of the next generation. They are not restored to the normal amount.

The presence of CenH3 in sperm has previously been demonstrated in mammals and Xenopus [43]–[45]. Similarly, the absence of Cenp-C in sperm has been observed in Xenopus [45]. A future analysis of the mechanism that selectively maintains all or at least a substantial amount of centromeric CenH3 during the radical chromatin re-organization that accompanies genome compaction into sperm heads will be of interest. The fact that CenH3 nucleosomes are not exchanged for protamines, in contrast to bulk nucleosomes, is crucial, at least in case of Drosophila sperm where centromeric Cid is an essential component of an epigenetic centromere mark for paternal chromosome maintenance in progeny. The demonstration that Cid is indispensible for epigenetic centromere marking in sperm may appear trivial in the light of the clear evidence that Cid is required and sufficient for centromere maintenance during mitotic proliferation [10],[21],[22]. However, recent findings in *C. elegans* [11],[12] and *A. thaliana* [13] have indicated that functional gametes do not necessarily require centromeric CenH3.

While the large majority of progeny generated after Cid elimination in sperm are gynogenetic haploid embryos, a fraction appears to have an extra chromosome with normal centromeric Cid levels. We cannot rule out that these near-haploid embryos represent cases where normal Cid amounts have been restored postzygotically on a particular paternal chromosome at the original centromere or at an ectopic location. The successful production of human artificial chromosomes (HACs), for example, is a clear case for de novo CenH3 acquisition and subsequent maintenance [20]. While the alpha-satellite arrays used in HAC production are completely CenH3-free before transfection, the centromeres in Cid-depleted sperm might have residual Cid below the level of detection in our experiments. A partial Cid depletion might also explain the apparently normal chromosome segregation during the two meiotic divisions. These meiotic divisions reduce Cid intensity per spot by a factor of at least four (Figure 1c) and thereby in our deGradFP experiments perhaps below our detection limit. Alternatively, it is not excluded that Cid depletion continues after the meiotic divisions in these deGradFP experiments. However, even if the near-haploid embryos were to result from postzygotic restoration after partial or complete Cid elimination in sperm, such centromere restorations would be rare exceptions and not the rule. Since postzygotic replenishment is not even effective after far more moderate Cid reduction in sperm by RNAi, we consider centromere restoration to be an unlikely explanation for the observed near-haploid embryos. Perhaps these embryos arise after missegregation of maternal chromosomes during the first embryonic mitoses because occasionally the lagging paternal chromosomes might affect the function of the gonomeric spindle. Consistent with this interpretation, embryos fathered by Cid-EGFP-depleted sperm often displayed a reduced and irregular nuclear density during the syncytial stages within the anterior region where fertilization occurs (33% versus 5% in controls). Similarly, polar body morphology in this anterior region was also often abnormal (64% versus 20% in controls). It appears therefore that the lagging paternal chromosomes somehow cause local cell cycle defects in a considerable fraction of the progeny.

The fact that centromeric Cid, after moderate reduction in sperm to 33%–50% of its normal level, is not restored back to normal during development of progeny with normal levels of maternal and zygotic *cid* expression demonstrates that the pre-existing level of centromeric Cid is a major determinant for quantitative control over centromeric Cid levels during cell cycle progression. Some restoration occurs within one generation according to our data, and Cid on the Y centromere no longer seems to be significantly reduced in spermatocytes of grandsons and great-grandsons of fathers with Cid-depleted sperm (N.R. and C.F.L., preliminary observations). However, it is clear that the efficiency of this restoration is poor. Starting from sperm, generation of F1 spermatocytes requires more than 2 wk of development, including progression through about 20 or more cell cycles. This is insufficient to replenish centromeric Cid to the normal level. Thus our data clearly support the idea that the Cid nucleosomes, which remain after random partitioning of pre-existing centromeric Cid nucleosomes onto the two sister chromatids during chromosome replication, instruct the local loading of an equivalent amount of new CenH3 nucleosomes during each cell cycle. Accordingly, centromeric Cid nucleosomes might be licensed for loading in a first cell cycle period, followed by actual loading and concomitant license consumption during a later cell cycle period. Overproduction of Cid and its loading factor Cal1 might by-pass the license requirement. Thus, the proposed quantitative dependence of Cid loading on pre-existing amounts is not necessarily incompatible with our finding that a centromeric Cid increase can be induced.

Apart from the fact that pre-existing centromeric Cid is critical for quantitative regulation, our overexpression experiments and the effects of *cid-GFP* transgene dose indicate that the level of *cid* expression is also a critical factor. We demonstrate that a single copy of this transgene under control of the *cid* cis-regulatory region (in a *cid* mutant background with Cid-EGFP as the only Cid source) is not sufficient for maintenance of centromeric Cid-EGFP at the level established in the presence of two copies. Therefore, the normal level of *cid* expression does not seem to be in great excess over what is required for centromere maintenance.

Our previous analyses have clearly revealed cell-cycle-dependent control of centromeric Cid deposition [9],[36]. In syncytial Drosophila embryos, Cid loading occurs during and depends on exit from mitosis. Studies in vertebrates [46]–[49] have similarly suggested that Cid loading in animal cells might generally depend on exit from M phase and that it occurs early in the cell cycle. However, here we demonstrate that cell-cycle coupling of Cid loading is subject to developmental control. Exit from M phase during the meiotic divisions in testis is not accompanied by Cid loading and expression of the loading factor Cal1. Instead, we observe Cal1-dependent loading during G2 before the onset of the meiotic divisions. Similarly, recent data have suggested that Cid loading in cultured Drosophila cells occurs already during metaphase (i.e., before exit from M phase) [50]. Moreover, observations from plant and fungal cells [51]–[53] have also indicated that the control of CenH3 loading during eukaryotic cell cycle progression is not governed by an invariant universal mechanism. Although presently precluded by background problems, a precise quantitative understanding of Cid loading throughout female gametogenesis would be of great interest.

The quantitative control of centromeric Cid during male and female gametogenesis might not be precisely identical and subtly subvert the quantitative control exerted by pre-existing Cid. Our quantification of centromeric Cid on Y, X, and autosomes is clearly consistent with the notion that centromeres are somewhat overloaded during passage through the male germline. This might explain the fact that the Y centromere, which is transmitted exclusively through the male germline, has about 2-fold higher levels of centromeric Cid. Moreover, the X centromere, which is transmitted more frequently through the female germline than any other centromere, seems to have the lowest amount of centromeric Cid. A possible reason for the postulated sex-specific difference in Cid loading might be linked to the fact that paternal centromeres experience exit from meiotic M phase not only in the testis but also again in the egg after fertilization during completion of female meiosis. Indeed we find that maternal Cid associates with paternal centromeres very early after fertilization during completion of the meiotic divisions of the oocyte. Importantly, mathematical analysis (Text S1) demonstrates that if the extent of over- and underloading are equal in the male and female germline, respectively, then a stable difference between Cid levels on paternal and maternal autosomal centromeres is reached within only two generations. Such a difference is also required for compatibility of our quantitative measurements (Figure 7g) with the parsimonious interpretation that centromeric Cid levels on autosomes (where we cannot assign parental origin) behave in the same way as revealed by our results concerning X and Y (where parental origin is known). Our mathematical analysis also implies that overloading in the male germline will result in a continuous increase of Y-centromeric Cid in the absence of counterbalancing mechanisms. In the case of the Y chromosome, Cid underloading in the female germline will of course not act as a counterbalancing process, but we speculate that the observed limited level of Cid expression might be involved. In addition, the Drosophila Y centromere contains unique telomere-related satellite repeats [54] that may have chromosome-specific effects. Even though centromeres in animals are specified primarily in an epigenetic manner, centromeric and pericentromeric DNA sequences are unlikely to be irrelevant and they have been implicated in meiotic drive and speciation [55].

Some aspects of centromere control that we have defined in Drosophila are presumably not valid or of minor importance in case of humans. In contrast to Drosophila, the Y centromere in human cell lines appears to have the lowest level of centromeric Cenp-A, while the X has average amounts [56]. Cenp-A levels on a given chromosome might vary considerably within the human population and appear to correlate with the size of the alpha-satellite region [57].

While our experiments concur with the notion that limited variation in the level of centromeric Cid is not necessarily detrimental, we also demonstrate that the variation of centromeric Cid on different chromosomes correlates with the amount of recruited kinetochore proteins, as previously found in some [58],[59] but not all experiments [60] with fungi. Moreover, evidence from human cancer cells has implicated Cenp-A overexpression in chromosome mis-segregation [61],[62]. Further clarification of the mechanisms that control centromeric CenH3 levels can therefore be expected to provide important insights into evolution of rogue cells, as well as of new species.

## Materials and Methods

### Drosophila Genetics

Most of the mutant alleles and transgenes used here have been characterized previously. *cid^T12-1^* and *cid^T22-4^* [22] carry premature stop codons. *cid^G5950^* (Bloomington Drosophila Stock Center #29695) has a P element insertion within the coding sequence. Moreover also *Cenp-C^prl41^* [63], *cal1^MB04866^* [9], and *Spc25^c00064^* [38] are known or predicted to abolish the production of gene products that can localize to centromeres. The transgenes *P{w^+^, gcid-EGFP-cid}III.2* [36], *P{w^+^, giEGFP-Cenp-C}II.1* [64], *P{w^+^, gi2xtdTomato-Cenp-C}II.3* and *III.1* [65], *P{w^+^, gcal1-EGFP}II.2* [9], and *P{w^+^, gSpc25-EGFP} II.1* [38] have been shown to complement recessive lethal mutations in the corresponding endogenous loci, demonstrating the functionality of the encoded fluorescently tagged centromere and kinetochore proteins. *P{w^+^, His2Av-mRFP}II.2* [36] and *P{w^+^, pUASt-mCherry-nls}III* were used for genotype marking in some experiments. *P{w^+^, pUASt-cal1}III.1* [9] was used for ectopic *cal1* expression.

The *C(1;Y), y^1^ v^1^ f^1^ B^1^: y^+^/C(1)RM, y^2^ su(wa)^1^ w^a^* stock for generation of X/0 males was kindly provided by Terry Orr-Weaver (Whitehead Institute for Biomedical Research, Cambridge, MA, USA). *P{w^+^,bamP-GAL4-VP16}III* [66], *P{w^+^,UASt-NSlmb-vhh-GFP4} III* [32], and *P{w^+^, Cid-RNAi^GD4436^}v43857* were kindly provided by D. McKearin, E. Caussinus, and the Vienna Drosophila RNAi Center (VDRC), respectively.

The *P{w^+^, gtopi-GAL4-VP16 }III* line was obtained by PhiC31-mediated germline transformation with pattB-topi-GAL4-VP16-topi. In this construct, the cis-regulatory sequences of the spermatocyte-specific gene *matotopetli* (*topi*) [67] control the production of a Gal4-VP16-Topi fusion protein. The *topi* cis-regulatory sequences were isolated by enzymatic DNA amplification with the primers NT15 (5′-CTTG GGATCC CTCGCAGATCGAATGTCTTG-3′) and NT16 (5′-CTTC AGATCT TTTCATGGCGCTAGTCCGAT-3′), the *GAL4-VP16* sequences with the primers NT17 (5′-CGACC AGATCT ATGAAGCTACTGTCTTCTATCG-3′), and NT19 (5′-GTTTA GCGGCCGC CCCACCGTACTCGTCAATTC-3′) from a bamP-GAL4-VP16 plasmid (kindly provided by D. McKearin), and the *topi* coding and 3′UTR sequences with NT20 (5′-AAGAG GCGGCCGCG ATGAAAGTCAAAGTTTCGGG-3′) and NT21 (5′-AATTC GCGGCCGC CGCTATCTTGCCGCTTTATTT-3′).

The *UAS-Cid-EGFP* lines were obtained after germline transformation with a pUAST construct where the sequences coding for Cid with an internal EGFP insertion were inserted after enzymatic amplification using pCaSpeR4-*gcid-EGFP-cid* [36] as a template in combination with the primers NT41 (5′-CTTTAA GCGGCCGC TTAAGCAAATACCGAAAATTTG-3′) and NT42 (5′-GCAAA TCTAGA AACTAAGCCTAAACTTCTCTTTTGG-3′).

The *UAS-cal1^RNAi^* lines were obtained after PhiC31-mediated germline transformation with a Valium20 [68] construct with an insert generated by annealing the oligonucleotides 5'-ctagcagt ACGAGTGTAGTTGCTGCAATA tagttatattcaagcata TATTGCAGCAACTACACTCGT gcg-3′ and 5′-attcgc ACGAGTGTAGTTGCTGCAATA tatgcttgaatataacta TATTGCAGCAACTACACTCGT actg-3′.

The testis squash preparations for the quantification of EGFP signals at centromeres and kinetochores were made with males of the following genotypes: w*; cid^T12-1^/cid^T22-4^; P{w^+^, gcid-EGFP-cid}III.2 (Figure 1b,c, Figure 4a, Figure 6a); w*; P{w^+^, giEGFP-Cenp-C}II.1; FRT82B Cenp-C^prl41^ (Figure 1b,d, Figure 6c); w*; P{w^+^, gSpc25-EGFP}II.1; Spc25^c00064^ (Figure 6d); and w*; P{w^+^, gcal1-EGFP}II.2; cal1^MB04866^ (Figure 4b, Figure S2).

Males with the first two genotypes were also crossed to *w^1118^* females for the analysis of the transmission of paternal centromere proteins in progeny embryos (Figure 2). Moreover, females with these genotypes were crossed to *w^1118^* males for the analysis of the association of maternally derived centromere proteins with sperm DNA (Figure 5). The squash preparations for the quantification of EGFP signals at centromeres and kinetochores of mitotic chromosomes (Figure 6e) were made with 1–2-h embryos collected from parents with the first three genotypes.

For deGrad Cid-EGFP during spermatogenesis (Figure 3), we generated w*; cid^T12-1^/cid^G5950^, P{w^+^, gcid-EGFP-cid}II.1; P{w^+^, UASt-NSlmb-vhhGFP4}III/P{w^+^, gtopi-GAL4-VP16-topi}III, P{w^+^, gcid-EGFP-cid}III.2 males by standard crossing schemes. In parallel, we generated w*; cid^T12-1^/cid^G5950^, P{w^+^, gcid-EGFP-cid}II.1; +/P{w^+^, gtopi-GAL4-VP16-topi}III, P{w^+^, gcid-EGFP-cid}III.2 males for control experiments. The males were crossed with w*; cid^T12-1^/cid^T22-4^; P{w^+^, gcid-EGFP-cid}III.2 females for analysis of the subsequent generation.

For the analysis of X/0 spermatocytes, we used testis isolated from *v^+^*, *f^+^*, *B^+^* males obtained after crossing *C(1;Y), y^1^ v^1^ f^1^ B^1^: y^+^* males with either w*; cid^T12-1^/cid^T22-4^; P{w^+^, gcid-EGFP-cid}III.2 (Figure 6b) or w*; P{w^+^, gSpc25-EGFP}II.1; Spc25^c00064^ females (Figure 6d).

To increase Cid-EGFP levels on sperm centromeres (Figure 7a,b), we generated w*; cid^T12-1^/cid^G5950^, P{w^+^, gcid-EGFP-cid}II.1; P{w^+^, pUASt-cal1}III.1, P{w^+^, pUASt-cid-EGFP-Cid} III.1/P{w^+^, bamP-GAL4-VP16}III, P{w^+^, gcid-EGFP-cid}III.2 males by standard crossing schemes. In parallel, w*; cid^T12-1^/cid^G5950^, P{w^+^, gcid-EGFP-cid}II.1; +/P{w^+^, bamP-GAL4-VP16}III, P{w^+^, gcid-EGFP-cid}III.2 males were generated for control experiments. For analysis in the next generation (Figure 7b), the males were crossed to w*; cid^G5950^, P{w^+^, gcid-EGFP-cid}II.1/cid^G5950^, P{w^+^, gi2xtdTomato-Cenp-C}II.3; P{w^+^, gcid-EGFP-cid} III.2/Cenp-C^prl41^, P{w^+^, gi2xtdTomato-Cenp-C}III.1 females.

To decrease Cid-EGFP levels on sperm centromeres (Figure 7c–g), we generated w*; cid^T12-1^/cid^G5950^, P{w^+^, gcid-EGFP-cid}II.1; P{w^+^, cid-RNAi^GD4436^}v43857/P{w^+^, bamP-GAL4-VP16}III, P{w^+^, gcid-EGFP-cid}III.2 males. In parallel, w*; cid^T12-1^/cid^G5950^, P{w^+^, gcid-EGFP-cid}II.1; +/P{w^+^, bamP-GAL4-VP16}III, P{w^+^, gcid-EGFP-cid}III.2 males were generated for control experiments. For analyses during embryogenesis of the next generation (Figure 7d), the males were crossed to w*; cid^G5950^, P{w^+^, gcid-EGFP-cid}II.1/cid^G5950^, P{w^+^, gi2xtdTomato-Cenp-C}II.3; P{w^+^, gcid-EGFP-cid} III.2/Cenp-C^prl41^, P{w^+^, gi2xtdTomato-Cenp-C}III.1 females. For analyses with wing imaginal discs of the next generation (Figure 7e), the males were crossed to w*; cid^T12-1^, P{w^+^, His2Av-mRFP}II.2/CyO, Dfd-EYFP females. Wing discs of larvae with *His2Av-mRFP* expression were mounted and imaged [9]. The rest of the larvae was used for further genotype analysis by PCR using primers specific for the *bam-GAL4-VP16* transgene and the P insertion in *cid^G5950^*, respectively. The data shown in Figure 7e are from the genotype w*; cid^G5950^, P{w^+^, gcid-EGFP-cid}II.1/cid^T12-1^, P{w^+^, His2Av-mRFP}II.2; {w^+^, bamP-GAL4-VP16}III, P{w^+^, gcid-EGFP-cid}III.2/+. We point out that this genotype, which does not include the *cid-RNAi^GD4436^* transgene, results from crosses with both the experimental and the control males. The data obtained with this genotype therefore cannot be affected by *cid-RNAi^GD4436^* expression during zygotic development. As shown in Figure S5c,d, data from additional progeny genotypes were fully consistent with the findings made with the genotype displayed in Figure 7e. For analyses with testis of the next generation (Figure 7f,g), the males were crossed to *P{w^+^, pUASt-mCherry-nls}III* females followed by isolation of testis from male progeny with the genotype w*; cid^G5950^, P{w^+^, gcid-EGFP-cid}II.1/+; P{Cid-RNAi^GD4436^}v43857/P{w^+^, pUASt-mCherry-nls}III or w*; cid^G5950^, P{w^+^, gcid-EGFP-cid}II.1/+; +/P{w^+^, pUASt-mCherry-nls}III in case of the control experiments. These testes were characterized by the presence of green centromeric signals and absence of red nuclear signals.

### Testis Preparations

Testis squash preparations were made, fixed, and stained essentially as described [69] with the following modifications. After dissection in testis buffer (183 mM KCl, 47 mM NaCl, 10 mM Tris-HCl, pH 6.8), testes were transferred to a 5 µl drop of phosphate buffered saline (PBS) on a poly-l-lysine-treated slide and cut open to spill the contents. The sample was squashed very gently after addition of 15 µl of 4% formaldehyde in PBS under a 22×22 mm siliconized cover slip. Fixation was continued for 6 min.

Testes whole mount immunolabeling was done as described [70] with the following modifications. After testis dissection (see above), fixation was done in 4% formaldehyde in PBS for 10 min. Antibody incubations were performed in a humid chamber.

For immunolabeling, rabbit antiserum against ModC [41] was diluted 1∶4,000 in PBS. Affinity-purified rabbit antibodies against Cenp-C [63] were diluted 1∶5,000. Hybridoma supernatant containing mouse monoclonal antibody eya10H6 (generated by S. Benzer and N.M. Bonini and kindly provided by the Developmental Studies Hybridoma Bank developed under the auspices of the NICHD and maintained by The University of Iowa, Department of Biology, Iowa City, IA 52242) or 38F3 against NopI/Fibrillarin (Abcam, ab4566) was diluted 1∶100 and 1∶300, respectively, Secondary antibodies were Cy5 or Alexa568-conjugated goat antibodies against rabbit or mouse IgG.

The images shown in Figure 1b, Figure 3a, Figure 4a,b, Figure S2, and Figure 6a,b represent projections of image stacks assembled using Adobe Photoshop. Deconvolution was performed before maximum projection in case of Figure 3a. To reveal the weaker signals in advanced stages of spermatogenesis, increasing adjustment of brightness and contrast was applied to the progressive stages shown in Figure 1b. Therefore, the EGFP signals displayed in Figure 1b do not reflect their quantified intensities (Figure 1c,d). However, to document differences between Cid-EGFP and Cenp-C-EGFP intensities, images were treated equally at a given stage. Concerning X/0 spermatocytes, we point out that the Cid-EGFP signals of the X and the fourth chromosomes were often tightly associated in a single cluster during S5 (in 65% of the spermatocytes, *n* = 25). The data displayed in Figure 6b were obtained from spermatocytes with separate X and chromosome 4 signals.

### Embryo Preparations

For analyses during the very early embryonic stages, eggs were collected for 30 min at 25°C. For analyses during the syncytial blastoderm cycles, eggs were collected for 1 h and aged for an additional hour. For analyses of nuclear densities during cellularization, the embryos were aged for an additional 1.5 h. After dechorionation, embryos were fixed and released from the vitelline membrane by shaking in methanol. After DNA staining with Hoechst 33258 (1 µg/ml in PBS), we mounted the embryos under a coverslip in 70% glycerol, 1% n-propyl gallate, and 0.05% p-phenylenediamine.

For the preparation of mitotic chromosome spreads, eggs were collected for 1 h and aged for an additional hour. Embryos were dechorionated in 1.4% sodium hypochlorite and extensively rinsed with deionized water. After transfer into an Eppendorf tube containing a 1∶1 mixture of heptane and Schneider’s tissue culture medium with 10 µM demecolcine (Sigma D7385), embryos were incubated on a rotating wheel. In case of the analysis of Cenp-EGFP and Spc25-EGFP, the incubation in demeocolcine was omitted. After 30 min, embryos were transferred to 75 mM KCl in a depression slide and incubated for 10 min. Embryos were then transferred into a 5 µl drop of polyamine buffer [71] on a glass slide and torn apart using fine tungsten needles. A drop of 5 µl of 4% formaldehyde in PBS was added. After addition of a coverslip, the sample was inverted onto a filter paper and squashed for a few seconds to spread the embryos. After a 5-min incubation, the sample was frozen in liquid nitrogen. After flipping away the coverslip, the slide was immediately placed into chilled 100% ethanol and incubated for 10 min at −20°C. Excess ethanol was removed by tapping the slide onto a paper towel. After washing the sample area with PBS for 5 min, DNA staining was performed with 0.5 µg/ml Hoechst 33258 in PBS for 10 min. After a 5-min wash in PBS, the sample was mounted under a coverslip in 70% glycerol, 1% n-propyl gallate, and 0.05% p-phenylenediamine.

Immunostainings of eggs and embryos shown in Figures 2 and 5 was performed as described [72]. Briefly, embryos were dechorionated in bleach, fixed in methanol, and rehydrated in 1× PBS, 0.15% Triton X-100. Embryos were then incubated overnight in the same buffer with rabbit anti-GFP antibody (Invitrogen) at a 1∶200 dilution. They were then washed three times in 1× PBS, 0.15% Triton X-100, and incubated overnight in secondary antibody (AlexaFluor 488 goat anti-rabbit (Molecular Probes) at 1∶1,000). After an incubation step in a RNAse A solution (2 mg/ml in PBS) for 1 h at 37°C, embryos were mounted in a mounting medium (DAKO) containing propidium iodide (5 µg/ml) to stain DNA. Male and female pronuclei at the pronuclear apposition stage and during the first prometaphase were identified based on their position. As previously revealed by immunolabeling using an antibody against actetylated histone H4, a histone mark that is enriched in paternal chromatin, the female pronucleus (or the maternal set of chromosomes) is known to be systematically oriented toward the polar bodies [27]. Accordingly, the first pronucleus encountered along the virtual line from polar bodies to the apposed pronuclei was considered to be the female pronucleus.

### Microscopy and Image Analysis

Quantification of EGFP signals on centromeres and kinetochores was performed after acquiring stacks (20–28 sections, 250 nm spacing) from squashed testis preparations using a 63×/1.4 oil immersion objective on a Zeiss Cell Observer HS microscope. Stacks were converted into maximum projections using ImageJ. Signal quantification was performed essentially as described previously [9] with the following modifications. For quantification of centromeric signal intensities during spermatogenesis, all centromeric signals within a cell were surrounded with the free hand tool followed by measurement of area (A_s_) and integrated pixel intensity (I_s_) of the selected regions. For subtraction of diffuse signals (background and GFP signals from any noncentromeric pools), the selected region was slightly enlarged yielding A_l_ and I_l_. Total centromeric signal intensity per cell was then calculated as I_s_−[A_s_×(I_l_−I_s_)/(A_l_−A_s_)]. An analogous subtraction of diffuse signals was performed for quantification of intensities of individual centromeres in spermatocytes where each centromeric spot was surrounded individually. The characteristics of the DNA staining pattern during the S5 spermatocytes stage provided the basis for an assignment of Cid-EGFP signals to different chromosomes. While the centromeres of the two chromosome 4 homologs in the large majority of all S5 spermatocytes analyzed are paired into a single Cid-EGFP spot next to a strongly staining DNA dot, each homolog of all the other chromosomes usually displays a single Cid-EGFP dot. The X centromere Cid-EGFP signal is usually also close to a region of intense DNA labeling, which however is more irregular in shape and not as intense as in case of chromosome 4. In contrast, the Y centromere is very rarely associated with a region of intense DNA staining presumably as a result of the Y loops present during the S5 stage. Finally, the territories of chromosome 2 and 3 display a far more homogenous DNA staining than the regions with chromosomes X, Y, and 4. We would like to point out that quantification of centromeric signals obtained after immunofluorescent labeling with anti-Cid, anti-Cenp-C, or anti-GFP resulted in far more noisy data. Moreover, comparison of GFP fluorescence signals and immunofluorescent signals after double labeling of cells expressing only Cid-EGFP or Cenp-C-EGFP with antibodies recognizing these GFP fusions indicated that immunofluorescent signal variability is likely to be caused by problems with antibody accessibility that at least in part also reflect the kinetochore attachment status. Accurate centromere signal quantification in combination with DNA fluorescent in situ hybridization (FISH) for chromosome identification was therefore not an option, also because GFP fluorescence does not survive the FISH procedure.

In case of the analyses in syncytial blastoderm embryos, stack size was 16 focal planes with 250 nm spacing, in case of wing discs, 20 focal planes with 250 nm spacing. For all quantitative analyses of EGFP signal intensities, data were acquired from at least three different slides. The data displayed in Figure 7b,d are from embryos in prometaphase or metaphase of mitosis 11 and 12. As we did not observe significant intensity differences between mitosis 11 and 12, values were pooled for preparation of the s.

## Supporting Information

Figure S1 Gynogenetic embryos resulting from Cid depletion in sperm progress through an additional syncytial cycle before cellularization. During spermatogenesis, a GFP-specific ubiquitin ligase [32] was either expressed (*+ deGrad cid-EGFP*) or not expressed (− *deGrad cid-EGFP*) in males producing only Cid-EGFP instead of normal Cid. Males were crossed with wild-type females, and progeny was fixed at the stage of cellularization. Comparison of the nuclear density in − and + *deGrad cid-EGFP* progeny during cellularization revealed a 2-fold higher value (or rarely a mosaic of regions with normal and 2-fold higher values) in the latter. Scale bar, 5 µm.
(TIF)

Figure S2 *cal1-EGFP* expression during spermatogenesis. Squash preparation of testis producing only Cal1-EGFP instead of endogenous Cal1 was stained for DNA and double labeled with antibodies against Cenp-C (CenpC) and Fibrillarin (Fibrillarin) to mark centromeres and nucleolus, respectively. Stacks of representative cells during the gonial division cycles (gonial) and during the spermatocyte stages S1 (S1), S3 (S3), and S5 (S5) were deconvolved and maximum projected. Cal1-EGFP dots co-localizing with Cenp-C were detected up to the S3 stage but not later. Cal1-EGFP signals could not be detected in the nucleolus, in contrast to the findings in embryonic and cultured Drosophila cells [9],[37]. Scale bar, 10 µm.
(TIF)

Figure S3 Comparison of Cid levels in different Y centromeres. (a) Crossing scheme for the introgression of different Y chromosomes into the *cid; cid-EGFP* background. The mini-*w^+^* gene of *P{w^+^, gcid-EGFP-cid}III.2* and the recessive mutation *curled* (*cu*) were used as marker mutations. (b) Squash preparation of testis with introgressed Y chromosome from strains *w^1^* (*w^1^*), *Oregon R* (*o*), *Thurgau 1* (*t*), *Winterthur 1* (w), *Congo* (*c*), *India* (*i*), or *Zimbabwe* (*z*). Cid-EGFP levels on individual centromeres were measured, indicating that all the different Y centromeres have similarly increased Cid levels in comparison to the other centromeres. The intensity of individual Cid-EGFP dots in S5 stage spermatocytes representing either a chromosome 2 or 3 centromere (2/3), the paired chromosome 4 centromeres (4p), the X centromere (X), or the Y centromere (Y) was measured, and the sum of all the individually measured centromeric signals within each analyzed spermatocyte was set to 100%. Bars indicate average relative intensity; s.d. is indicated by whiskers. *n*>25. The isofemale strains *Thurgau 1* and *Winterthur 1* were established from single females isolated from the wild at different locations in Switzerland in spring 2010 (P. Radermacher, L. Baumann, and C.F. Lehner., unpublished). The strains *Congo* (*c*), *India* (*i*), and *Zimbabwe* (*z*) were kindly provided by G. Reuter (University of Halle, Halle, Germany).
(TIF)

Figure S4 Effect of gene dose on centromeric Cid-EGFP levels. (a) Wing imaginal discs expressing *cid-EGFP* were isolated from wandering third instar larvae and imaged [9]. The larvae had either one endogenous *cid^+^* gene copy and one *cid-EGFP* transgene copy (*cid^−/+^; cid-EGFP*) or no endogenous *cid^+^* gene copy and either two (*cid* ^−/−^; *cid-EGFP/cid-EGFP*) or one (*cid* ^−/−^ *; cid-EGFP/+*) transgene copy. Scale bar, 10 µm. (b) Total Cid-EGFP signal intensity per nucleus was measured in cells of the peripodial membrane of wing imaginal discs from the different genotypes (as in a). Bars represent average intensity in arbitrary units (a.u.), with whiskers indicating s.d. A similar number of cells was analyzed in each disc. The total number of cells and imaginal discs analyzed is given below the bars (*n*). According to *t* test, differences between the analyzed genotypes were highly significant (*p*<0.0001). (c) Comparison of Cid-EGFP levels in individual centromeres of Y (Y), X (X), major autosomes (2/3), and the paired chromosome 4 centromeres (4p) in spermatocytes of *cid* males with two or one copy of *cid-EGFP*, as indicated. Major autosome territories contain two spots. The stronger (s) and weaker (w) spots, respectively, were grouped and analyzed separately. Dots indicate centromeric EGFP intensity in arbitrary units (a.u.). Averages (long horizontal line) are given with s.d. (short horizontal lines). *n*>45. The fold change of average Cid-EGFP levels between samples with two or one cid-EGFP copy is indicated. All the indicated differences were highly significant according to *t* test (*p*<0.0001).
(TIF)

Figure S5 Transgenerational maintenance after Cid-EGFP reduction in sperm. (a, b) Analysis of the extent of Cid-EGFP knock-down during spermatogenesis. Centromeric Cid-EGFP signals were quantified in males without (−) or with (+) *bamP-GAL4-VP16*-driven expression of *UAS-Cid^RNAi^* in a background producing only Cid-EGFP instead of endogenous Cid. (a) Centromeric Cid-EGFP levels per nucleus were quantified in S5 spermatocytes, spermatids, and sperm. The extent of average reduction of centromeric Cid-EGFP resulting from RNAi is indicated above the brackets and was found to be highly significant in all cases (*p*<0.0001, *t* test). At least 25 cells from at least five different testes were analyzed for each stage and genotype. (b) Centromeric Cid-EGFP levels in individual centromeres of Y (Y), X (X), major autosomes (2/3), and the paired chromosome 4 centromeres (4p) were quantified in S5 spermatocytes. Each major autosome territory contains two Cid-EGFP spots. The stronger (s) and weaker (w) spots, respectively, were grouped and analyzed separately. The extent of average reduction of centromeric Cid-EGFP resulting from RNAi is indicated above the brackets and was found to be highly significant in all cases (*p*<0.0001, *t* test). At least 35 centromeres from at least five different testes were analyzed for each case. (c, d) Analysis of propagation of reduced centromeric Cid-EGFP levels in the next generation. Centromeric Cid-EGFP per nucleus in progeny derived from males without (−) or with (+) RNAi-mediated Cid-EGFP reduction in sperm (as determined in Figure 7c and Figure S5, a and b) was compared. In peripodial cells of wing imaginal discs of third instar larvae, centromeric Cid-EGFP levels were measured before genotype assignment by PCR. While data from the genotype w*; cid^G5950^, P{w^+^, gcid-EGFP-cid}II.1/cid^T12-1^, P{w^+^, His2Av-mRFP}II.2; {w^+^, bamP-GAL4-VP16}III, P{w^+^, gcid-EGFP-cid}III.2/+ are displayed in Figure 7e, further corroborating data from the genotypes w*; cid^T12-1^/cid^T12-1^, P{w^+^, His2Av-mRFP}II.2; {w^+^, bamP-GAL4-VP16}III, P{w^+^, gcid-EGFP-cid}III.2/+ (c) and w*; cid^G5950^, P{w^+^, gcid-EGFP-cid}II.1/cid^T12-1^, P{w^+^, His2Av-mRFP}II.2; P{w^+^, cid-RNAi^GD4436^}v4385 or +/+ (d) are shown here. The fold change of average Cid-EGFP levels between controls and experimental samples is indicated next to the dashed arrows. Statistical significance of the changes according to *t* test: *p*<0.001 (d) and *** *p*<0.0001 (c). The total number (*n*) of analyzed cells and imaginal discs analyzed is given below the bars. Dots indicate centromeric EGFP intensity per nucleus (a, c, d) or in individual centromeres (b) in arbitrary units (a.u.) chosen to result in an average intensity of 100 a.u. in control spermatocytes in (a), (c), and (d). Averages (long horizontal line) are given with s.d. (short horizontal lines).
(TIF)

Text S1 A deterministic model for sex-specific differences of Cid loading on autosomes.
(PDF)

## Abbreviations

Cal1: chromosome alignment 1 protein
CenH3: centromere-specific histone H3 variant
Cenp-A: centromere protein A
Cenp-C: centromere protein C
Cid: centromere identifier
EGFP: enhanced green fluorescent protein
