# Supplementary figures and images for: Transgenerational Propagation and Quantitative Maintenance of Paternal Centromeres Depends on Cid/Cenp-A Presence in Drosophila Sperm
Source: PLoS Biol. 2012 Dec 27;10(12):e1001434. doi: 10.1371/journal.pbio.1001434 (PMC3531477; doi:10.1371/journal.pbio.1001434)

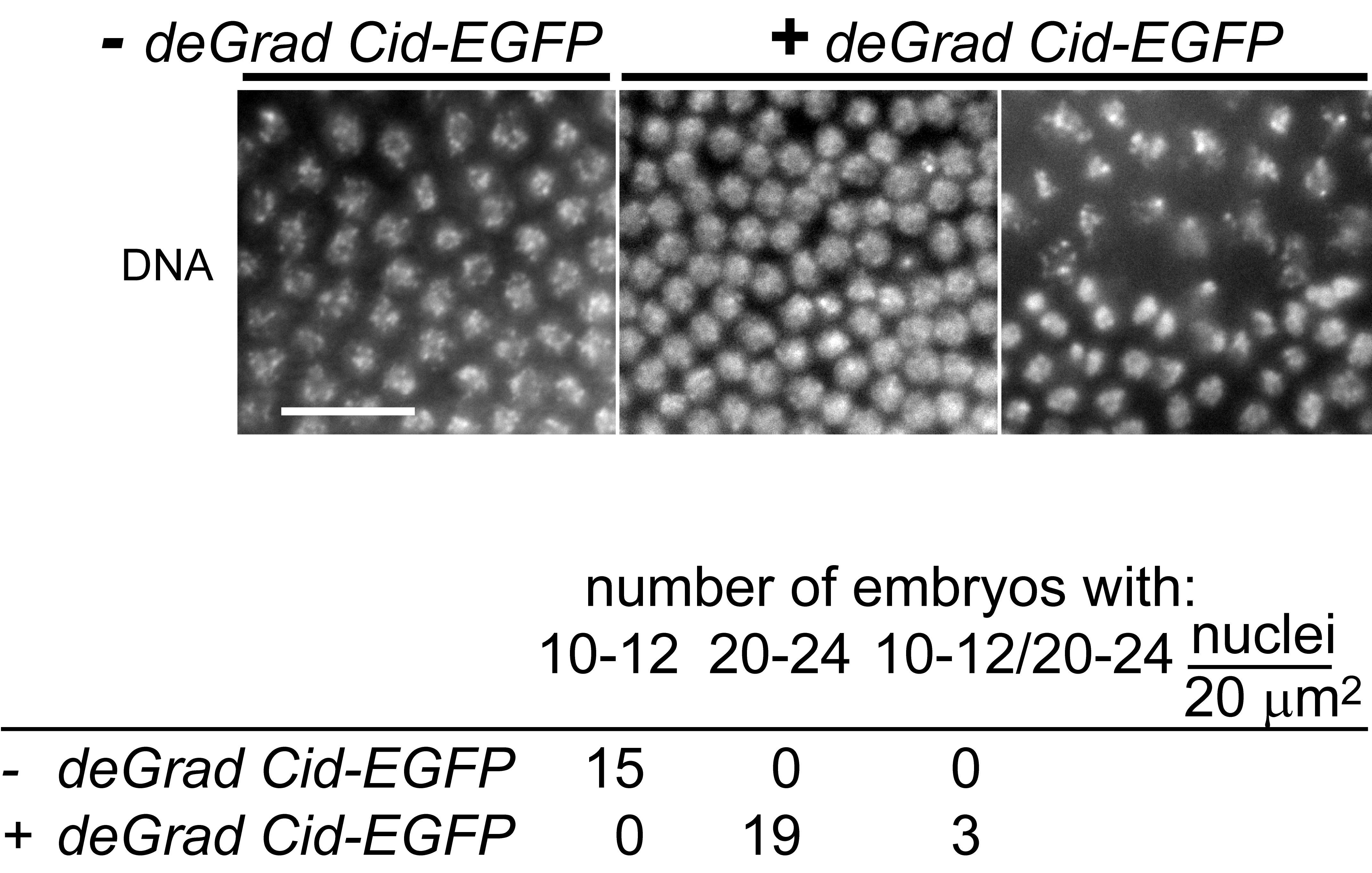

Supplement: Figure S1 — Gynogenetic embryos resulting from Cid depletion in sperm progress through an additional syncytial cycle before cellularization. During spermatogenesis, a GFP-specific ubiquitin ligase [32] was either expressed (+ deGrad cid-EGFP) or not expressed (− deGrad cid-EGFP) in males producing only Cid-EGFP instead of normal Cid. Males were crossed with wild-type females, and progeny was fixed at the stage of cellularization. Comparison of the nuclear density in − and + deGrad cid-EGFP progeny during cellularization revealed a 2-fold higher value (or rarely a mosaic of regions with normal and 2-fold higher values) in the latter. Scale bar, 5 µm. (TIF) [file pbio.1001434.s001.tif]

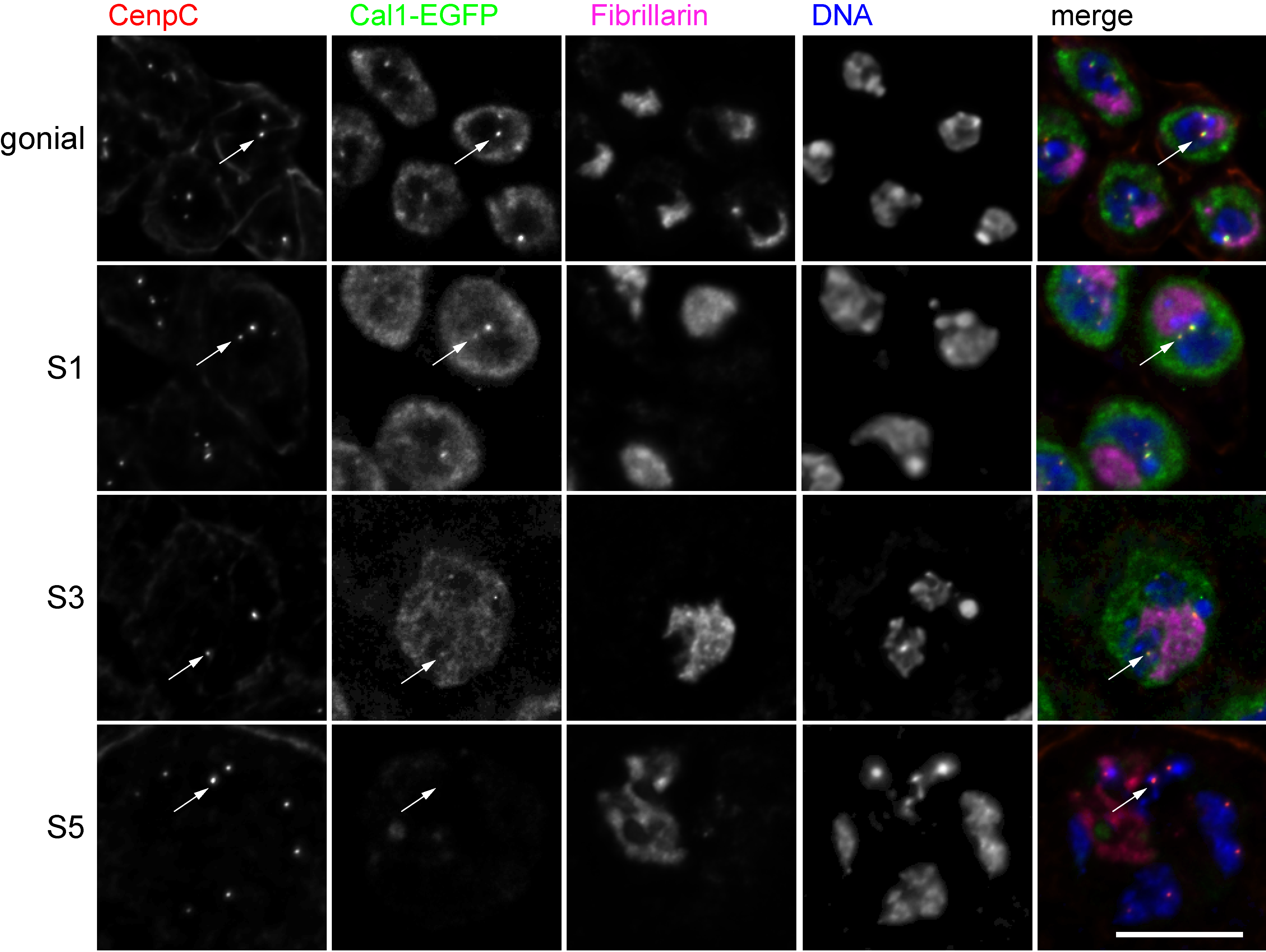

Supplement: Figure S2 — cal1-EGFP expression during spermatogenesis. Squash preparation of testis producing only Cal1-EGFP instead of endogenous Cal1 was stained for DNA and double labeled with antibodies against Cenp-C (CenpC) and Fibrillarin (Fibrillarin) to mark centromeres and nucleolus, respectively. Stacks of representative cells during the gonial division cycles (gonial) and during the spermatocyte stages S1 (S1), S3 (S3), and S5 (S5) were deconvolved and maximum projected. Cal1-EGFP dots co-localizing with Cenp-C were detected up to the S3 stage but not later. Cal1-EGFP signals could not be detected in the nucleolus, in contrast to the findings in embryonic and cultured Drosophila cells [9],[37]. Scale bar, 10 µm. (TIF) [file pbio.1001434.s002.tif]

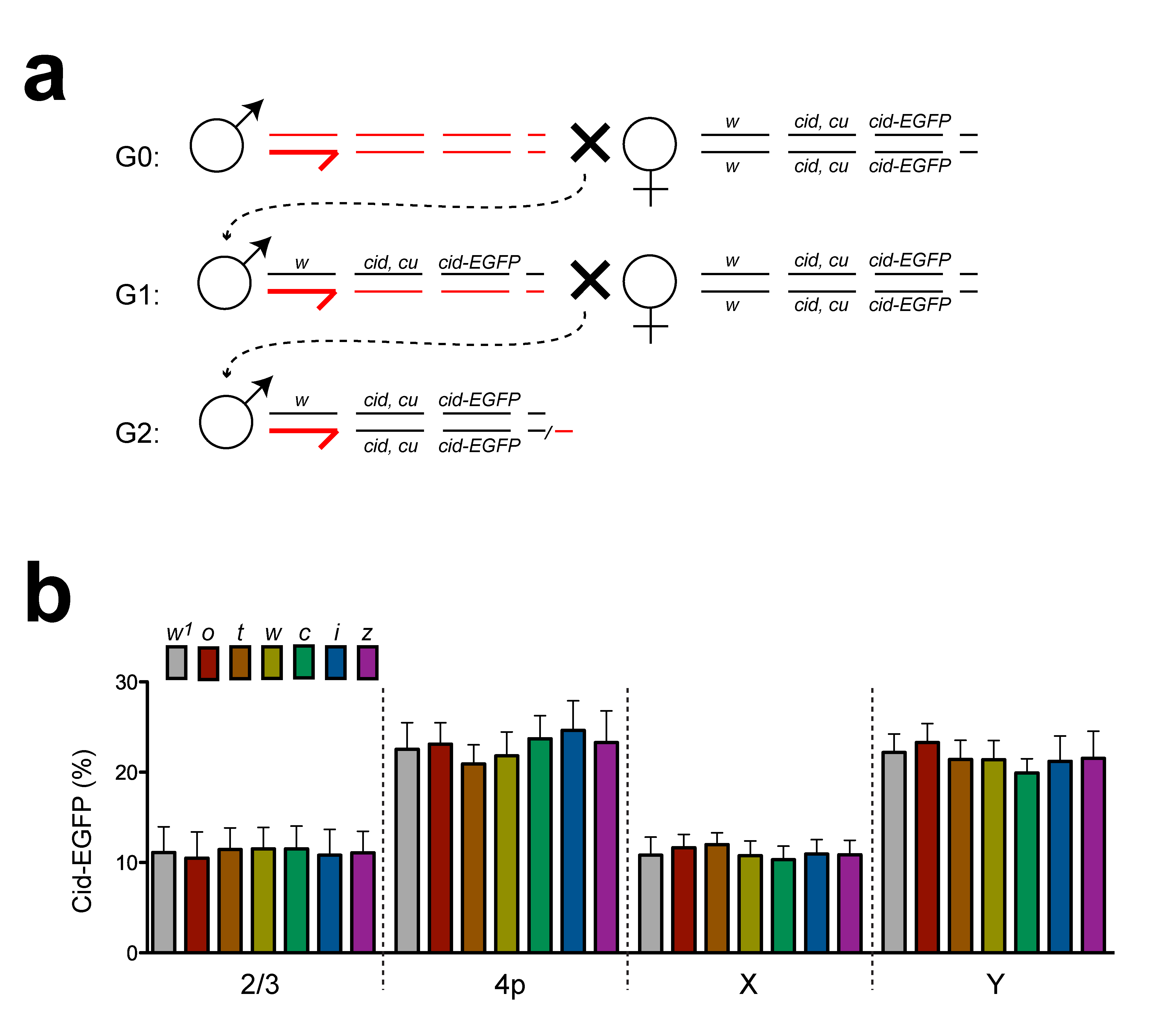

Supplement: Figure S3 — Comparison of Cid levels in different Y centromeres. (a) Crossing scheme for the introgression of different Y chromosomes into the cid; cid-EGFP background. The mini-w+ gene of P{w+, gcid-EGFP-cid}III.2 and the recessive mutation curled (cu) were used as marker mutations. (b) Squash preparation of testis with introgressed Y chromosome from strains w1 (w1), Oregon R (o), Thurgau 1 (t), Winterthur 1 (w), Congo (c), India (i), or Zimbabwe (z). Cid-EGFP levels on individual centromeres were measured, indicating that all the different Y centromeres have similarly increased Cid levels in comparison to the other centromeres. The intensity of individual Cid-EGFP dots in S5 stage spermatocytes representing either a chromosome 2 or 3 centromere (2/3), the paired chromosome 4 centromeres (4p), the X centromere (X), or the Y centromere (Y) was measured, and the sum of all the individually measured centromeric signals within each analyzed spermatocyte was set to 100%. Bars indicate average relative intensity; s.d. is indicated by whiskers. n>25. The isofemale strains Thurgau 1 and Winterthur 1 were established from single females isolated from the wild at different locations in Switzerland in spring 2010 (P. Radermacher, L. Baumann, and C.F. Lehner., unpublished). The strains Congo (c), India (i), and Zimbabwe (z) were kindly provided by G. Reuter (University of Halle, Halle, Germany). (TIF) [file pbio.1001434.s003.tif]

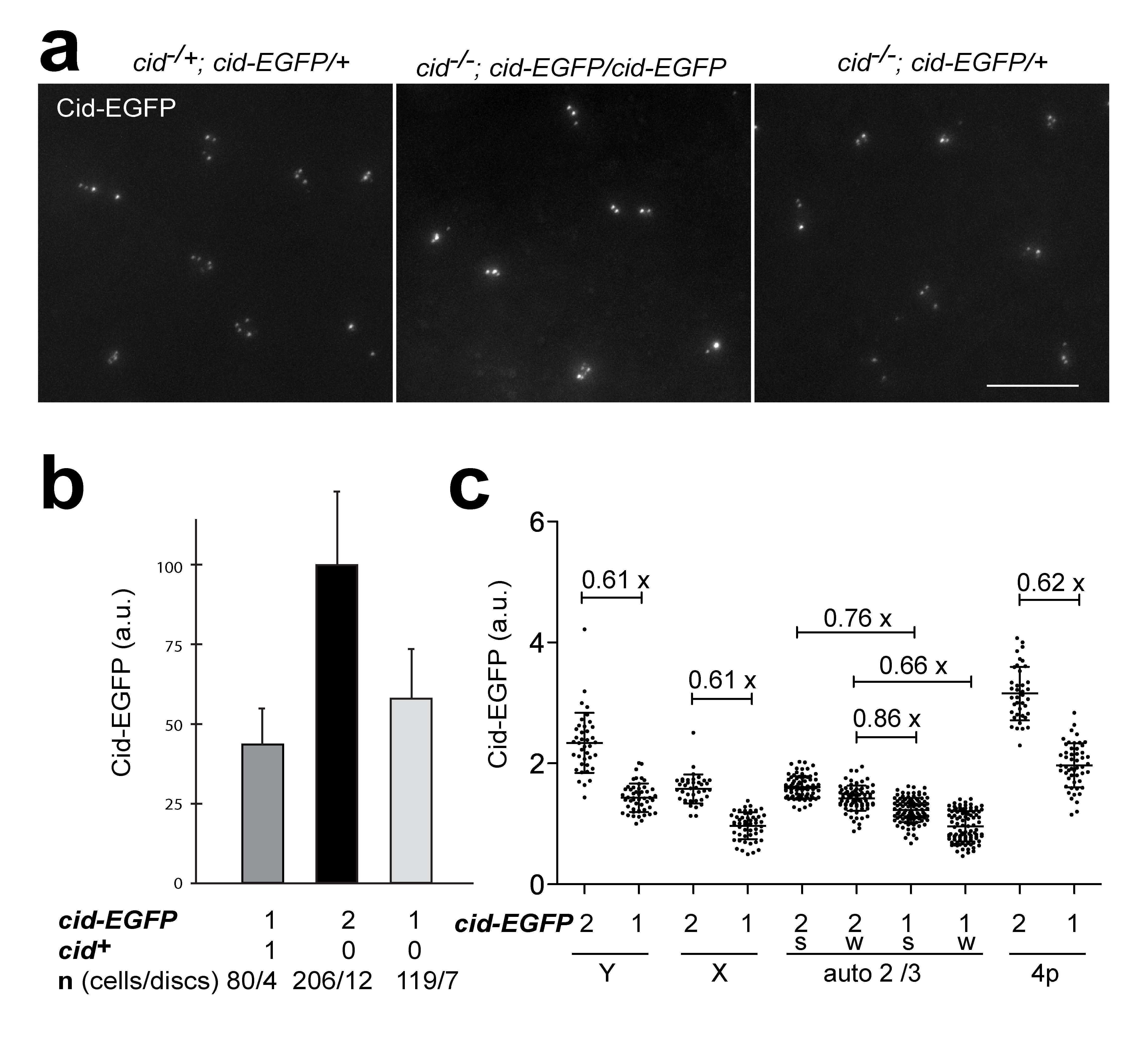

Supplement: Figure S4 — Effect of gene dose on centromeric Cid-EGFP levels. (a) Wing imaginal discs expressing cid-EGFP were isolated from wandering third instar larvae and imaged [9]. The larvae had either one endogenous cid+ gene copy and one cid-EGFP transgene copy (cid−/+; cid-EGFP) or no endogenous cid+ gene copy and either two (cid −/−; cid-EGFP/cid-EGFP) or one (cid −/− ; cid-EGFP/+) transgene copy. Scale bar, 10 µm. (b) Total Cid-EGFP signal intensity per nucleus was measured in cells of the peripodial membrane of wing imaginal discs from the different genotypes (as in a). Bars represent average intensity in arbitrary units (a.u.), with whiskers indicating s.d. A similar number of cells was analyzed in each disc. The total number of cells and imaginal discs analyzed is given below the bars (n). According to t test, differences between the analyzed genotypes were highly significant (p<0.0001). (c) Comparison of Cid-EGFP levels in individual centromeres of Y (Y), X (X), major autosomes (2/3), and the paired chromosome 4 centromeres (4p) in spermatocytes of cid males with two or one copy of cid-EGFP, as indicated. Major autosome territories contain two spots. The stronger (s) and weaker (w) spots, respectively, were grouped and analyzed separately. Dots indicate centromeric EGFP intensity in arbitrary units (a.u.). Averages (long horizontal line) are given with s.d. (short horizontal lines). n>45. The fold change of average Cid-EGFP levels between samples with two or one cid-EGFP copy is indicated. All the indicated differences were highly significant according to t test (p<0.0001). (TIF) [file pbio.1001434.s004.tif]

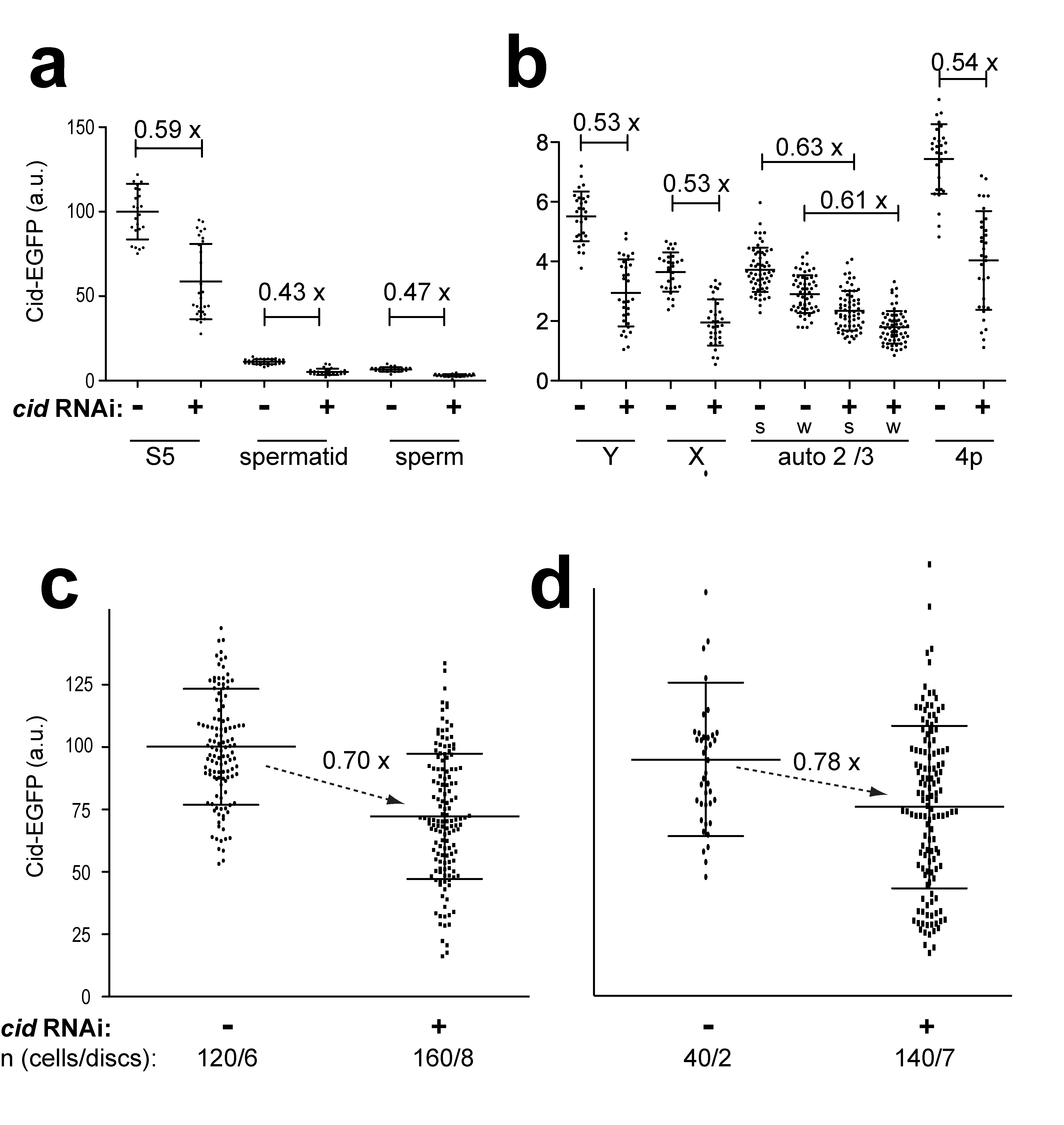

Supplement: Figure S5 — Transgenerational maintenance after Cid-EGFP reduction in sperm. (a, b) Analysis of the extent of Cid-EGFP knock-down during spermatogenesis. Centromeric Cid-EGFP signals were quantified in males without (−) or with (+) bamP-GAL4-VP16-driven expression of UAS-CidRNAi in a background producing only Cid-EGFP instead of endogenous Cid. (a) Centromeric Cid-EGFP levels per nucleus were quantified in S5 spermatocytes, spermatids, and sperm. The extent of average reduction of centromeric Cid-EGFP resulting from RNAi is indicated above the brackets and was found to be highly significant in all cases (p<0.0001, t test). At least 25 cells from at least five different testes were analyzed for each stage and genotype. (b) Centromeric Cid-EGFP levels in individual centromeres of Y (Y), X (X), major autosomes (2/3), and the paired chromosome 4 centromeres (4p) were quantified in S5 spermatocytes. Each major autosome territory contains two Cid-EGFP spots. The stronger (s) and weaker (w) spots, respectively, were grouped and analyzed separately. The extent of average reduction of centromeric Cid-EGFP resulting from RNAi is indicated above the brackets and was found to be highly significant in all cases (p<0.0001, t test). At least 35 centromeres from at least five different testes were analyzed for each case. (c, d) Analysis of propagation of reduced centromeric Cid-EGFP levels in the next generation. Centromeric Cid-EGFP per nucleus in progeny derived from males without (−) or with (+) RNAi-mediated Cid-EGFP reduction in sperm (as determined in Figure 7c and Figure S5, a and b) was compared. In peripodial cells of wing imaginal discs of third instar larvae, centromeric Cid-EGFP levels were measured before genotype assignment by PCR. While data from the genotype w*; cidG5950, P{w+, gcid-EGFP-cid}II.1/cidT12-1, P{w+, His2Av-mRFP}II.2; {w+, bamP-GAL4-VP16}III, P{w+, gcid-EGFP-cid}III.2/+ are displayed in Figure 7e, further corroborating data from the genotypes w*; cidT12-1/cidT12-1, [file pbio.1001434.s005.tif]
