# Supplementary material for: Transgenerational Propagation and Quantitative Maintenance of Paternal Centromeres Depends on Cid/Cenp-A Presence in Drosophila Sperm
Source: PLoS Biol. 2012 Dec 27;10(12):e1001434. doi: 10.1371/journal.pbio.1001434 (PMC3531477; doi:10.1371/journal.pbio.1001434)
Supplement: Text S1 — A deterministic model for sex-specific differences of Cid loading on autosomes. (PDF) [file pbio.1001434.s006.pdf]

Text S1: A deterministic model for sex-specific differences of Cid loading on autosomes

We formulate a simple deterministic model of differential Cid loading onto autosomes in the male and female germline. We then test the consequences of the model assumptions on the expected mean Cid content of chromosomes in a population at equilibrium.

Assuming non overlapping generations and a 1:1 sex ratio, for any generation one can envisage a population chromosome pool that can be subdivided into four categories as described below. For each category, we assign a mean Cid level designated by the variables:

M_m : mean Cid level on chromosomes of maternal origin in males,

M_p : mean Cid level on chromosomes of paternal origin in males,

F_m : mean Cid level on chromosomes of maternal origin in females,

F_p : mean Cid level on chromosomes of paternal origin in females.

Consider M_m and M_p at time *t.* If one makes the simplifying assumption that for the next generation at *t+1*, the Cid level on chromosomes of paternal origin will increase by a proportion *d*, and the Cid level on chromosomes of maternal origin will decrease by *d*, then one can formulate a set of difference equations where

$$M_m(t+1) = \tfrac{1}{2}(1-d)\,F_m(t) + \tfrac{1}{2}(1-d)\,F_p(t) = \tfrac{1}{2}(1-d)\,(F_m(t) + F_p(t)) \qquad \text{(eq. 1)}$$

$$M_p(t+1) = \tfrac{1}{2}(1+d)\,M_m(t) + \tfrac{1}{2}(1+d)\,M_p(t) = \tfrac{1}{2}(1+d)\,(M_m(t) + M_p(t)). \qquad \text{(eq. 2)}$$

Similarly,

$$F_m(t+1) = \tfrac{1}{2}\,(1\text{-}d)\,(\,F_m(t) + F_p(t)\,) \qquad \text{(eq. 3)}$$

$$F_p(t+1) = \tfrac{1}{2}\,(1+d)\,(\,M_m(t) + M_p(t)\,). \qquad \text{(eq. 4)}$$

Given the above system (eqs. 1 to 4), one sees that regardless of initial conditions, for any time t, after one generation we have

$$M_m(t+1) = F_m(t+1) \qquad \text{(eq. 5)}$$

$$M_p(t+1) = F_p(t+1). \qquad \text{(eq. 6)}$$

Given eqs. 1,2,5 and 6, in generation $t+2$ we have

$$M_m(t+2) = \tfrac{1}{2}\,(1\text{-}d)\,(\,F_m(t+1) + F_p(t+1)\,) = \tfrac{1}{2}\,(1\text{-}d)\,(\,M_m(t+1) + M_p(t+1)\,) \qquad \text{(eq. 7)}$$

$$M_p(t+2) = \tfrac{1}{2}\,(1+d)\,(\,M_m(t+1) + M_p(t+1)\,). \qquad \text{(eq. 8)}$$

Using eqs. 5 and 6, we also know that $F_m(t+2) = M_m(t+2)$ and $F_P(t+2) = M_P(t+2)$. Hence, using the values in eqs. 7 and 8, and the recursion from eq. 1, the expression for M_m at generation $t+3$ is

$$M_m(t+3) = \tfrac{1}{2}\,(1\text{-}d)\,(\,F_m(t+2) + F_p(t+2)\,) = \tfrac{1}{2}\,(1\text{-}d)\,(\,M_m(t+2) + M_p(t+2)\,)$$

$$= \tfrac{1}{2}\,(1\text{-}d)\,(\,\tfrac{1}{2}\,(1\text{-}d)\,(\,M_m(t+1) + M_p(t+1)\,) + \tfrac{1}{2}\,(1+d)\,(\,M_m(t+1) + M_p(t+1)\,)\,)$$

$$= \tfrac{1}{2}\,(1\text{-}d)\,(\,M_m(t+1) + M_p(t+1)\,). \qquad \text{(eq. 9)}$$

Similarly,

$M_p(t+3) = ½\,(1+d)\,(\,M_m(t+2) + M_p(t+2)\,)$

$= ½\,(1+d)\,(\,M_m(t+1) + M_p(t+1)\,).$ (eq. 10)

Examining equations 7 to 10, we see the notable result that $M_m(t+3) = M_m(t+2)$ and $M_p(t+3) = M_p(t+2)$. Hence, regardless of initial conditions, the system will reach an equilibrium after 2 generations. For any $n>2$, the equilibrium Cid contents are given by

$M_m(t+n) = ½\,(1-d)\,(\,M_m(t+1) + M_p(t+1)\,)$

$= ¼\,(1-d)\,(\,(1-d)\,(\,F_m(t) + F_p(t)\,) + (1+d)\,(\,M_m(t) + M_p(t)\,)\,)$ (eq. 11)

and

$M_p(t+n) = ½\,(1+d)\,(\,M_m(t+1) + M_p(t+1)\,)$

$= ¼\,(1+d)\,(\,(1-d)\,(\,F_m(t) + F_p(t)\,) + (1+d)\,(\,M_m(t) + M_p(t)\,)\,).$ (eq. 12)

Given that $M_m(t+n)=F_m(t+n)$ and $M_p(t+n)=F_p(t+n)$, we hence have expressions for the equilibrium Cid content of all male and female chromosome categories. Note that when at the starting conditions the Cid content of all categories have the same value x, such that $F_m(t) = F_p(t) = M_m(t) = M_p(t) = x$, then eqs. 11 and 12 reduce to

$M_m(t+n) = (1-d)\,x$ (eq. 13)

and

$M_p(t+n) = (1+d)\,x.$ (eq. 14)

The results expressed in equations 11 to 14 show that if one assumes a symmetrical overloading/underloading proportion *d*, then after two generations the population will reach an equilibrium where the ratio of maternal to paternal Cid levels is given by

$$\frac{Mm(t+n)}{Mp(t+n)} = \frac{1-d}{1+d}\,. \qquad \text{(eq. 15)}$$

The same ratio is also valid for the female autosomal chromosomes.

The interpretation of eq. 15 is that after two generations, the system will maintain a constant underload of Cid on maternally derived chromosomes, and a constant overload on the paternally derived chromosomes.

Definining *f* as the ratio between maternal and paternal Cid levels in eq. 15, we have

$f = (1-d) / (1+d)$

and

$d = (1 - f) / (1 + f).$

If the average of the weaker and stronger Cid-EGFP signal in each autosome territory in control spermatocytes as determined (Figure 7g) were to correspond to Mm and Mp, respectively, *f* would amount to about 0.8. Accordingly, under- and overloading in the female and male germline, respectively, would be predicted to change Cid-EGFP levels by 11% in

each generation ($d = 0.11$).
